# Adequate post-ischemic reperfusion of the mouse brain requires endothelial NFAT5

**DOI:** 10.1186/s40478-024-01918-5

**Published:** 2024-12-22

**Authors:** Reiner Kunze, Paul Wacker, Paula Breuer, Emil Nasyrov, Ivan M. Kur, Andreas Weigert, Andreas H. Wagner, Hugo H. Marti, Thomas Korff

**Affiliations:** 1 Institute of Physiology and Pathophysiology, Department of Cardiovascular Physiology, Heidelberg University, Heidelberg, Germany; 2 Centre for Ophthalmology, University Eye Hospital Tuebingen, Tuebingen, Germany; 3 Institute of Biochemistry I, Faculty of Medicine, Goethe University Frankfurt, 60590 Frankfurt am Main, Germany; 4 European Center for Angioscience (ECAS), Medical Faculty Mannheim, Heidelberg University, 69120 Heidelberg, Germany

**Keywords:** NFAT5, KCNJ2, Endothelial cells, Ischemic stroke, Reperfusion

## Abstract

Severity and outcome of strokes following cerebral hypoperfusion are significantly influenced by stress responses of the blood vessels. In this context, brain endothelial cells (BEC) regulate inflammation, angiogenesis and the vascular resistance to rapidly restore perfusion. Despite the relevance of these responses for infarct volume and tissue recovery, their transcriptional control in BEC is not well characterized. We revealed that oxygen and nutrient-deprived BEC activate nuclear factor of activated T-cells 5 (NFAT5)—a transcription factor that adjusts the cellular transcriptome to cope with environmental stressors. We hypothesized that NFAT5 controls the expression of genes regulating the response of BEC in the ischemic brain. The functional relevance of NFAT5 was assessed in mice, allowing the conditional EC-specific knock-out of *Nfat5* (*Nfat5*^*(EC)−/−*^). Cerebral ischemia was induced by transient middle cerebral artery occlusion (MCAO) followed reperfusion up to 28 days. While loss of endothelial *Nfat5* did not evoke any phenotypic abnormalities in mice under control conditions, infarct volumes, neurological deficits and the degree of brain atrophy were significantly pronounced following MCAO as compared to control animals (*Nfat5*^*fl/fl*^). In contrast, MCAO-induced edema formation, inflammatory processes and angiogenesis were not altered in *Nfat5*^*(EC)−/−*^ mice. RNAseq analyses of cultured BEC suggested that loss of NFAT5 impairs the expression of *Kcnj2* encoding a potassium channel that may affect reperfusion. In fact, lower levels of KCNJ2 were detected in arterial endothelial cells of *Nfat5*^*(EC)−/−*^ versus *Nfat5*^*fl/fl*^ mice. Laser speckle contrast imaging of the brain revealed an impaired perfusion recovery in *Nfat5*^*(EC)−/−*^ versus *Nfat5*^*fl/fl*^ mice after MCAO.Collectively, NFAT5 in arterial BEC is required for an adequate reperfusion response after brain ischemia that is presumably dependent on the maintenance of *Kcnj2* expression. Consequently, impairment of the protective role of endothelial NFAT5 results in enlarged infarct sizes and more severe functional deficits of brain functions.

**Supplementary Information:**

The online version contains supplementary material available at 10.1186/s40478-024-01918-5.

## Introduction

Ischemic stroke due to the occlusion of a main cerebral artery leads to a critical shortage of oxygen, glucose and further nutrients in the affected brain area, which initiates a complex cascade of several interrelated and overlapping detrimental events. This ischemic cascade encompasses initial bioenergetic failure and osmotic stress, followed by excitotoxicity, oxidative stress, dysfunction of the blood–brain barrier (BBB), and inflammation, which altogether lead to the progressive death of neurons, glial and endothelial cells [12, 23]. After onset of vessel occlusion, ischemic brain injury occurs when cerebral blood flow (CBF) drops below a critical level, resulting in irreversibly injured tissue with acute neuronal death (ischemic core). This core is surrounded by tissue where perfusion is severely reduced resulting in loss of neuronal function albeit still providing sufficient blood flow to allow neuronal cell survival. This severely ischemic tissue at risk is salvageable and is called the “penumbra”. If this “penumbra” area, however, is not rescued because CBF is not restored in time, it will progressively be recruited to the infarct core [5]. To emphasize the need for early treatment of stroke patients to reverse the ischemic process, the the term “time is brain” was coined. Accordingly, the penumbra is the target of acute ischemic stroke management, and the clinical goal is to restore CBF as soon as possible, as no proven reperfusion therapy exists beyond 24 h after onset of stroke [15].

However, hypoxia and oxidative stress not only initiate detrimental events leading to the death of brain-resident cells but they can also induce intrinsic stress responses to protect these cells from ischemic cell death and preserve their function. In response to low tissue oxygenation and oxidative stress, cells activate hypoxia-inducible factors (HIFs) and the redox-sensitive transcription factor NF-E2 related factor 2 (NRF2) that drive global genomic responses facilitating metabolic reprogramming, anti-apoptotic mechanisms, and antioxidant defense, respectively [48, 50]. Likewise, the stress-responsive transcription factor Nuclear factor of activated T-cells 5 (NFAT5 or tonicity enhancer binding protein (TonEBP) was shown to be relevant for coping with the environmental stress following ischemia. *Nfat5* haploinsufficient mice have been shown to exhibit more severe neurological deficits, larger infarct volumes, elevated BBB hyperpermeability and brain swelling as compared to their wild type littermates [34]. Furthermore, *Nfat5* deficiency strongly enhanced the vulnerability of neurons and astrocytes to ischemic stress conditions in vitro [34, 52].

NFAT5 is a calcineurin-independent member of the Rel family of transcription factors that regulates multiple types of cellular stress responses associated with hyperlipidemia, insulin resistance, biomechanical load, hypertension, arteriosclerosis, rheumatoid arthritis, hypertonicity and hypoxia [3, 9, 16, 22, 26, 36, 42, 49, 53]. Therefore, tissue hypoxia, osmotic stress, oxidative stress and/or inflammation evolving during ischemic stroke may also activate NFAT5-dependent stress responses in brain cells upon ischemic injury. Recently, we showed that hypoxia triggers the activity of NFAT5 [27] in endothelial cells, which play a critical role in the regulation of inflammation, tissue regeneration and brain (re)perfusion after ischemic stroke. We thus hypothesized that NFAT5 controls endothelial stress responses after ischemic stroke, which are required for tissue recovery. To test our hypothesis, we applied a transient cerebral ischemia model in mice, allowing for the inducible endothelial cell-specific knockout of *Nfat5* and evaluated the consequences of intermittent brain ischemia on the histological and functional level.

## Materials and methods

### Experimental animals

All transgenic mouse lines were established on a C57BL/6 background. We used female and male littermate mice that were age-matched between experimental groups. All animal experiments were approved by the local animal welfare committee (Regierungspräsidium Karlsruhe, Germany, permission number: 35–9185.81/G-251/19), conformed to the Guide for the Care and Use of Laboratory Animals published by the US National Institutes of Health and were performed in accordance with the Animal Research: Reporting In Vivo Experiments (ARRIVE) guidelines (https://www.nc3rs.org.uk/arrive-guidelines). Mice were housed at constant room temperature (22 ± 2 °C) and relative humidity (50–55%) on a controlled 12:12 h light–dark cycle and were provided with standard laboratory chow (LASQCdiet Rod16; LASvendi, Soest, Germany) and water ad libitum. *Nfat5*^*fl/fl*^ mice [25] were crossed with *Tg(Cdh5-cre/ERT2)*^*1Rha*^ mice [44, 51]. Mice were genotyped using primers (Eurofins Genomics, Ebersberg, Germany) described in **Table S1**. The genetic ablation of NFAT5 in the corresponding offspring (age: 5–7 weeks) was induced by application of 1 mg tamoxifen per day i.p. for 5 consecutive days (*Nfat5*^*(EC)−/−*^) or miglyol as solvent control (hereinafter referred to as *Nfat5*^*fl/fl*^). Mice were used in experiments after a recovery period of 3 weeks.

### Experimental stroke model

Mice were used at the age of 8–10 weeks. Mice were anesthetized by a mixture of 2% isoflurane, 70% N_2_O and remainder O_2_, and were maintained by reducing the isoflurane concentration to 1.0–1.5%. To induce focal cerebral ischemia a 7–0 silicon rubber-coated nylon monofilament (Doccol Corporation, Redlands, CA, USA) was introduced into the left internal carotid artery and pushed toward the left middle cerebral artery (MCA) as previously described [24]. The intraluminal suture was left for 45 min. Subsequently, animals were re-anesthetized and the occluding monofilament was withdrawn to allow reperfusion for 3–24 h and 28 days, respectively. For sham surgery, the mice underwent the same procedure without vessel occlusion. The animals were maintained at 37 °C during and after surgery until they fully recovered from anesthesia. Then, mice were returned to their solitary cages in a heated (30 °C) environment with free access to food and water for 12 h. During the remaining time animals were kept under normal conditions as described above. **Table S2** lists the criteria resulting in exclusion from end-point analysis.

Operators and investigators were blinded for the mouse genotype in all experiments and analyses. Evaluation of all read-out parameters was done independently and in a blinded fashion.

### Neurobehavioral analyses

A modified neurological severity score was assessed to grade the neurologic function on a scale of 0 to 14 (normal score 0; maximal deficit score 14) [8]. Motor coordination and balance were assessed by using the Rotarod performance test. Mice were placed individually on the revolving drum. Once they were balanced, the drum was accelerated from 4 to 40 revolutions per minutes over the course of 300 s, and the time at which the animal dropped off the drum was determined (maximum testing time 300 s). Mice were trained for three consecutive days (three runs each) before stroke and were tested at the indicated time points post-stroke (two runs each).

### Laser speckle contrast imaging (LSCI)

Cortical blood flow (CBF) was monitored using a two-dimensional laser speckle contrast analysis system (PeriCam PSI High Resolution with PIMSoft; Perimed, Stockholm, Sweden). Mice were anesthetized with isofluorane and maintained at physiological body temperature as described above. A midline incision was made in the scalp and the skull surface cleaned with sterile normal saline. A charged-coupled device camera was placed 9.5 cm above the skull using a Pericam PSI System and blood perfusion images were taken at 30 min after MCAO and at 3 h upon onset of reperfusion. Raw speckle images were taken in a 1.8 cm × 1.6 cm field (at 1 frame/s). Ten frames averaging, with the resolution of 0.02 mm six consecutive images at each time point per animal were averaged for analysis of CBF expressed in arbitrary units (a.u.). The entire ipsilateral and contralateral hemispheres were defined as the main region of interest (ROI). Across the ipsilateral hemisphere further ROIs were determined at 30 min post-MCAO based on the following blood flow thresholds: infarct core (CBF < 40% of mean contralateral hemisphere), hypoperfused peri-infarct region (CBF between 40 and 70% of mean contralateral hemisphere).

### *Drug application *in vivo

The thymidine analog bromodeoxyuridine (BrdU, Sigma-Aldrich, Steinheim, Germany, #B5002, 10 mg/ml PBS) was applied intraperitoneally at a dosage of 50 mg/kg once daily for four consecutive days from day 4 to 7 post-surgery. DyLight® 488 Lycopersicon Esculentum tomato lectin (Biozol, Calabasas, CA, USA, #DL-1174–1) at a dosage of 75 µg/mouse was injected into the mouse tail vein 60 min before sacrifice.

### Histopathological analyses

Animals were sacrificed by decapitation, brains were removed, and embedded into Tissue-Tek (Sakura Finetek, Staufen, Germany). From each brain, 24 coronal Sects. (10 µm thickness; 0.4 mm distance) were prepared using a Leica CM1520 cyrostat (Leica Biosystems, Wetzlar, Germany) at a constant temperature of − 15 °C, and stained with cresyl violet (#105,235, Merck Millipore, Darmstadt, Germany) according to manufacturer's instructions. Stained brain slices were digitized, and infarct and edema volume were measured using the image analysis software ImageJ (National Institutes of Health, Bethesda, MD, USA) as described previously [31, 40].

Neuronal degeneration was detected by performing Fluoro-Jade C (FJC) staining of coronal cerebral cryosections (10 µm thickness; + 0.74 mm relative to Bregma) following manufacturer's instructions. Briefly, cryosections were air-dried and fixed with 4% paraformaldehyde (PFA) in PBS (137 mM NaCl, 2.7 mM KCl, 10 mM Na_2_HPO_4_, 1.5 mM KH_2_PO_4_, pH 7.4–7.6) for 15 min at room temperature. Slices were dried on a slide warmer at 50 °C for 30 min followed by multiple immersion steps in 1% NaOH in 80% ethanol, 70% ethanol, dH_2_O and 0.06% KMnO_4_ (background blocking). Slices were incubated in 0.0001% Fluoro-Jade C (Merck Millipore, #AG325) in 0.1% acetic acid containing 1 µg/ml DAPI (Sigma-Aldrich, #D9542) for 20 min in the dark followed by multiple washing steps. Finally, slides were immersed in xylene for 1 min and mounted with Eukitt (Kindler, Bobingen, Germany). Sections were scanned by using a Zeiss Axiovert 200 M fluorescence microscope (Carl Zeiss Microscopy, Göttingen, Germany) equipped with a Plan-Apochromat 20x/0.8 objective for 20-fold magnification (Carl Zeiss Microscopy) and an Orca Flash 4.0 V2 digital camera (Hamamatsu Photonics, Herrsching am Ammersee, Germany). Image processing was performed by using TissueFAXS slides 4.2 software (TissueGnostics, Vienna, Austria) and exporting generated TIFF files. Analysis of acquired images was performed using FIJI software (National Institutes of Health). Allen mouse brain atlas (https://mouse.brain-map.org/static/atlas) served as reference to determine anatomical sub-regions. DAPI staining was used to identify Nuclei. FJC^+^ degenerated neurons per area were counted automatically and user-independent in the ipsilateral striatum, cortex and total hemisphere using FIJI software.

Cell apoptosis was quantified by using TdT-mediated dUTP nick end labeling (TUNEL). Coronal cerebral cryosections (10 µm; + 0.74 mm relative to Bregma) were fixed with 3% PFA in PBS for 15 min. Cryosections were incubated with 0.1% NaBH_4_/PBS for 15 min and permeabilized with 0.1% TritonX-100/PBS. TUNEL reaction mixture was prepared according to manufacturer's instructions with the following constitution: 1 × TdT reaction buffer, 2.5 mM CoCl_2_, 10 U/µl recombinant terminal transferase (Roche Diagnostics, Mannheim, Germany, #RTT-RO kit) and 0.01 mM Fluorescein-12-dUTP solution (Thermo Fisher Scientific, Dreieich, Germany, #R0101). Slices were incubated with TUNEL reaction mixture for 3 h at 37 °C. Nuclei were counterstained with 1 µg/ml DAPI for 10 min. Mowiol 4–88 (Merck Milipore, #475,904) was used to seal the stained cryosections. Image acquisition and processing were performed using the same equipment and software as described above. TUNEL^+^ apoptotic cells per area were counted automatically and user-independent in the ipsilateral striatum, cortex and total hemisphere using FIJI software.

### Immunofluorescence staining, image acquisition and analysis

Brain tissue slices and cell monolayer used for immunofluorescent staining were washed regularly between the indicated working steps with one of the following buffers: PBS, PBS-T (0.05% Tween-20 in PBS), TBS (50 mM Tris, 150 mM NaCl, pH 7.4–7.6) or TBS-T (0.05% Tween-20 in TBS).

Nuclear NFAT5 protein abundance or Kir2.1 protein levels in endothelial cells in vivo were determined by co-immunofluorescence staining of CD31 and NFAT5 (or Kir2.1). Immunofluorescence detection of alpha-smooth muscle actin (αSMA) was applied to discriminate between arteries and capillaries. Coronal cerebral cryosections (10 µm; + 0.74 mm relative to Bregma) were fixed with Zinc-fixative (100 mM Tris, 3.2 mM Ca(CH_3_COO)_2_, 22.8 mM ZnC_4_H_6_O_4_, 36.7 mM ZnCl_2_, pH 7.4) for 30 min at room temperature. Cryosections were permeabilized with 0.5% saponin (Sigma-Aldrich, #47,036) in PBS for 10 min. Slices were incubated for 60 min with blocking buffer (0.1% BSA, 0.25% casein in 50 mM Tris) followed by incubation with primary antibodies overnight at 4 °C and compatible fluorochrome-conjugated secondary antibodies for 1 h (for all antibodies see **Table S3**). All antibodies were diluted in blocking buffer. Nuclei were counterstained with 1 µg/ml DAPI for 10 min. Stained cryosections were sealed with Mowiol 4–88. Image acquisition and processing were conducted as described above. NFAT5^+^/CD31^+^/DAPI^+^ nuclei per area were determined manually in the peri-infarct zone of mice subjected to ischemia/reperfusion (I/R) injury and in the corresponding region of sham-operated animals. Automated determination of Kir2.1 levels in CD31^+^ EC were determined as described in Supplement S11 and S12.

Functional, perfused cerebrovasculature was detected across coronal cerebral cryosections by staining of intravenously applied DyLight® 488-conjugated tomato lectin in combination with CD31 immunofluorescence staining. Staining procedure was conducted as described above with the following modifications: (i) cryosections were blocked with 10% fish serum blocking buffer (Thermo Fisher Scientific, #37,527) in PBS-T and (ii) all antibodies were diluted in LowCross buffer (Candor Bioscience, Wangen, Germany, #100,125). Image acquisition and processing were performed as indicated above. The density of (i) CD31^+^, (ii) Lectin^+^ and (iii) CD31^+^/Lectin^+^ vessel segments was analyzed automatically and user-independent in the ipsi- and contralateral striatum, cortex and total hemisphere using FIJI software.

Cerebrovascular pericyte coverage was assessed by co-immunofluorescence staining of CD31 and PDGFRβ of coronal cerebral cryosections. The technical procedure was the same as described for the NFAT5/CD31 co-immunofluorescence staining.Image acquisition and processing were conducted as described above. FIJI software was used for the automatic and user-independent quantification of (i) CD31^+^, (ii) PDGFRβ^+^ and (iii) CD31^+^/PDGFRβ^+^ area in the ipsilateral striatum, cortex and total hemisphere. Cerebrovascular pericyte coverage was estimated by evaluating the ratio of PDGFRβ^+^/CD31^+^ area relative to total CD31^+^ area.

Proliferating endothelial cells in vivo were determined by co-immunofluorescence staining of BrdU and CD31. BrdU was applied intraperitoneally for labeling of proliferating cells as described above. Coronal cerebral cryosections were fixed with zinc-based fixative for 30 min, permeabilized with 0.5% saponin (Sigma-Aldrich, #47,036) in PBS for 10 min followed by 60 min incubation in 10% fish serum blocking buffer in PBS-T. Then, slices were incubated with anti-CD31/PECAM-1 antibody overnight at 4 °C followed by incubation with appropriate fluorophore-conjugated secondary antibody for 1 h. Nuclei were counterstained with 1 µg/ml DAPI for 10 min. Subsequently, sections were fixed with 4% PFA in PBS for 15 min, incubated in DNA denaturation buffer containing 1% Triton X-100 in 4 M HCl for 10 min followed by multiple washing steps with 100 mM sodium borate (pH 8.5) and PBS. Cryosections were blocked with 10% fish serum blocking buffer in PBS-T for 60 min followed by incubation with anti-BrdU antibody overnight at 4 °C and compatible fluorophore-conjuguated secondary antibody for 1 h. All antibodies were diluted in LowCross buffer. Stained cryosections were sealed with Mowiol 4–88. Image acquisition and processing were performed as described above. Proliferating endothelial cells were determined by automatic and user-independent quantification of BrdU^+^/CD31^+^/DAPI^+^ nuclei per area in the ipsilateral striatum, cortex and total hemisphere using FIJI software.

For immunofluorescence analyses of cultured brain endothelial cells (BEC), cells were fixed in ice-cold methanol for 15 min and allowed to dry for 20 min followed by rehydration. Cells were blocked with 10% donkey serum (anprotec, Bruckberg, Germany, #AC-SM-0101) and 0.5% Triton X-100 in PBS for 30 min followed by incubation with primary antibodies overnight at 4 °C and appropriate fluorophore-conjugated secondary antibodies for 2 h. All antibodies were diluted in blocking buffer. Nuclei were counterstained with 1 µg/ml DAPI for 15 min. Stained cells were sealed with Mowiol 4–88. Fluorescence images were recorded using a fluorescence microscope IX83 (Olympus, Hamburg, Germany). Nuclear NFAT5 protein abundance was determined by quantification of NFAT5 fluorescence intensity in NFAT5^+^/DAPI^+^ co-stained areas utilizing the cellSens Dimension Desktop software (v. 1.12, Olympus). Five fields of view (average intensity) per recorded well were included in the analysis.

### Flow cytometry

*Nfat5*^*(EC)−/−*^ and *Nfat5*^*fl/fl*^ mice were subjected to 45 min of MCAO followed by 24 h reperfusion. Sham-operated mice served as control. Single-cell suspensions were prepared from mouse brains from the ipsilateral brain hemisphere through mechano-enzymatic tissue digestion as described below. Flow cytometry was applied to determine number and polarization of brain-resident microglia and infiltrating leukocytes. Cells were analyzed using multicolor flow cytometry. Acquisition and data analysis were performed with a FACSymphony™ A5 SE (BD Biosciences, San Jose, CA, USA) in combination with FACSDiva software v9.0 (BD Biosciences) and FlowJo software v10 (Tree Star, Ashland, OR, USA). Before use, antibodies and reagents were titrated to ensure optimal concentrations. Brilliant Stain Buffer (BD Biosciences, #563,794) was added to each sample to prevent polymer–polymer interactions allowing efficient PMT voltage determination. Multi-color compensation matrices were created using single-color compensation beads (BD Biosciences, #552,845). Before staining, cells were incubated in anti-CD16/32 (Fc) blocking reagent (Miltenyi Biotec, Bergisch Gladbach, Germany, #130–092-575) for 10 min at 4 °C. Cells were stained with specific antibodies (summarized in Table S4) in staining buffer for 15 min at 4 °C in the dark. Then, Zombie UV™ Fixable Viability Kit (BioLegend, San Diego, CA, USA, #423,108) was added and samples were incubated for 10 min at 4 °C in the dark. Before the cells were acquired in the flow cytometer, cells were washed and counting beads (Bangs Laboratories, Fishers, IN, USA, #580) were added. The gating strategy was as follows: In the first step, living single cells were identified. Therefore, cell debris were removed via forward side (FSC)-A versus side scatter (SSC)-A gating followed by dead cell removal. FSC-A versus FSC-W gating was used to remove doublets. Then, subsets of CD45^+^ immune cells were analyzed. P2RY12 and CD49d expression were used to distinguish microglia (P2RY12^+^/CD49d^−^) from macrophages. F4/80 expression was analyzed in the microglia population. CD11b expression was used to distinguish myeloid from lymphoid cells. The CD11b^−^ population included T cells (CD11b^−^/CD3^+^) and B cells (CD11b^−^/CD19^+^). The CD11b^+^ population included neutrophils (CD11b^+^/Ly6G^+^), monocytes (CD11b^+^/Ly6C^+^/MHCII^−^) and macrophages (CD11b^+^/Ly-6C^+^/F4/80^+^/MHCII^−/+^). CD80 and CD206 expression were used as polarization markers in microglia and macrophages.

### Isolation of brain endothelial cells

Single-cell suspensions were prepared from ipsilateral brain hemispheres through mechano-enzymatic tissue digestion including neural tissue dissociation, myelin removal and erythrocyte lysis. Briefly, mice were sacrificed followed by transcardial perfusion. The right atrium was incised and mice were perfused with PBS at a rate of 2 ml/min for 5 min. Brains were harvested as described above and stored in Hanks' Balanced Salt Solution (HBSS, Sigma-Aldrich, #H8264). The weight of brain tissue was determined. Brains were transferred into a gentleMACS C Tube (Miltenyi Biotec, #130–093-237) containing pre-heated digestion mixture of the following constitution: 3 U/ml Dispase II (Sigma-Aldrich, #D4693) and 100 U/ml DNAse I (Roche Diagnostics, #11,284,932,001) in HBSS. Then, mechano-enzymatic tissue digestion was performed using a gentleMACS Dissociator (Miltenyi Biotec, #130–093-235) according to manufacturer's instructions followed by incubation on a tube rotator (4 rpm) at 37 °C for several times. Homogenate was pipetted up and down for 5–6 times using a 18 gauge needle and incubated once more on a tube rotator. Then, cell suspension was passed through a 70-µm cell strainer (Miltenyi Biotec, #130–110-916) placed on a 50 ml falcon containing 20% fetal bovine serum (FBS, Sigma-Aldrich, #10,270–106) in PBS. Cell suspension was centrifuged at 300xg for 10 min at room temperature. The supernatant was removed and the pellet was resuspended in 0.5% bovine serum albumin (BSA, Sigma-Aldrich, #A2153) in PBS. Myelin Removal Beads II (Miltenyi Biotec, #130–096-733) for magnetic labeling were added according to manufacturer's instructions followed by incubation for 15 min at 4 °C in the dark. After magnetic labeling, cells were washed by adding 10 × the labeling volume (0.5% BSA/PBS). Cell suspension was centrifuged at 300xg for 10 min at 4 °C. The supernatant was removed and the pellet was resuspended in 0.5% BSA/PBS. Next, cell suspension was passed through MACS LS Columns (Miltenyi Biotec, #130–042-401) placed in the magnetic field of a corresponding MACS Separator (Miltenyi Biotec, #130–042-302) according to manufacturer's instructions. Effluent consisting of unlabeled myelin-free cells was collected. Then, cell suspension was centrifuged at 300xg for 10 min at 4 °C. The supernatant was removed and the pellet was resuspended in ammonium–chloride–potassium (ACK) lysing buffer for red blood cell lysis of the following constitution: 0.15 M NH_4_Cl, 0.01 M KHCO_3_, 0.0001 M Na_2_EDTA, pH 7.2–7.4. Cells were incubated in ACK buffer for 5 min at room temperature. 5% FBS in PBS was added to stop lysis. Cell suspension was centrifuged at 300xg for 10 min at 4 °C. The pellet was resuspended in 5% FBS in PBS and used for flow cytometric analysis.

#### Primary brain endothelial cell culture

Microvascular BEC were prepared from adult mice (8–12 weeks) as described by [4] with some modifications. *Nfat5*^*fl/fl*^* X Tg(Cdh5-cre/ERT2)*^*1Rha*^ mice were sacrificed by cervical dislocation. Brains were removed, cerebra were prepared and stored in Dulbecco’s PBS (Thermo Fisher Scientific, #14,190,144) on ice. Meninges were removed using a cellulose chromatography paper (sterilized at 180 °C). Brains were transferred into a tissue grinder (Sigma-Aldrich, #D9938) containing working medium which consists of DMEM-F12 (Thermo Fisher Scientific, #21,331,020), 2 mM L-glutamine (Thermo Fisher Scientific, #24,020,091) and penicillin/streptomycin at a working concentration of 100 U/ml and 100 µg/ml (Thermo Fisher Scientific, #15,140,122). Brains were homogenized using two pistils. The homogenate was transferred into a centrifuge tube and centrifuged for 5 min at 1,350xg at 4 °C. The supernatant was removed using a vacuum pump. The pellet was resuspended in 18% dextran solution (MP Biomedicals, Santa Ana, CA, USA, #180,140) containing L-glutamine and penicillin/streptomycin at the same working concentrations as mentioned above. Dextran was dissolved in DPBS. The homogenate was vortexed extensively followed by centrifugation for 10 min at 6,080xg at 4 °C. After centrifugation, the myelin layer and dextran solution were removed. The pellet was resuspended in digestion medium containing DMEM (Thermo Fisher Scientific, #11,966,025), penicillin/streptomycin, 1 mg/ml collagenase/dispase (Roche Diagnostics, #11,097,113,001), 0.147 µg/ml Nα-Tosyl-L-lysine chloromethyl ketone hydrochloride (TLCK, Sigma-Aldrich, #90,182) and 4 µg/ml DNase I. The tissue was digested for 1 h 15 min in a 37 °C water bath followed by centrifugation for 5 min at 1,350xg at room temperature. The supernatant was removed and the pellet was resuspended in warm DPBS. The suspension was centrifuged once more for 5 min at 1,350xg at room temperature. DPBS was removed and the pellet was resuspended in full medium containing DMEM-F12, 20% Plasma-derived bovine serum (PDS, First Link, Wolverhampton, UK, #60–00-810), penicillin/streptomycin in combination with 0.25 µg/ml amphotericin B (antibiotic/antimycotic, Thermo Fisher Scientific, #15,240,062), 2 mM L-glutamine, 30 µg/ml Endothelial Cell Growth Supplement (ECGS, Sigma-Aldrich, #E2759) and 15 I.E./ml heparin (Ratiopharm, Ulm, Germany, # PZN003029843). Cell suspension was mixed carefully. Before the cell suspension was seeded, cell culture plates were coated with mouse collagen type IV (Corning Inc., Corning, NY, USA, #354,233) at a collagen concentration of 2.5 µg/cm^2^ diluted with 0.05 M hydrochloric acid followed by multiple washing steps with DPBS. Cell suspension was seeded in cell culture flasks or plates using 5–6 brains for T75 flasks. 8 µg/ml puromycin (Sigma-Aldrich, #P8833) was added to the full medium. The next day, cells were washed twice with DPBS containing calcium and magnesium (Thermo Fisher Scientific, #14,040,133) and fresh full medium containing 8 µg/ml puromycin was added. On the following day, full medium was changed without additional puromycin. After isolation, medium was changed twice a week. Culture was split 1:2 as soon as reaching confluence using 0.05% trypsin (Thermo Fisher Scientific, #25,300,054). Trypsin was inactivated with full medium. After splitting, cells were seeded on 6-well plates for further treatment.

#### *Nfat5 knockdown *in vitro

BEC from *Nfat5*^*fl/fl*^* X Tg(Cdh5-cre/ERT2)*^*1Rha*^ mice were isolated and cultured as described above. After first splitting, BEC were grown to 70–80% confluence. Cells were washed twice with DPBS containing calcium and magnesium. (Z)-4-hydroxytamoxifen (4-HT, Abcam, Cambridge, UK, #ab141943) was dissolved in DMSO and stored at a concentration of 100 mM at − 20 °C. The stock solution was diluted with full medium to a working concentration of 1 µM. BEC were exposed to 1 µM 4-hydroxytamoxifen or solvent (DMSO/full medium) for 3 days. After 4-HT treatment, cells were washed twice with DPBS as described above. BEC were cultured for a further 2–3 days before cells were used for experiments.

#### Oxygen–glucose deprivation (OGD)

BEC were washed twice with glucose-free HBSS consisting of (in mM): 1.0 CaCl_2_·2H_2_O, 5.0 KCl, 0.4 KH_2_PO_4_, 0.5 MgCl_2_·6H_2_O, 0.4 MgSO_4_·7H_2_O, 140 NaCl, 4.0 NaH-CO_3_ and 0.3 Na_2_HPO_4_·2H_2_O. The medium was pre-equilibrated in an Invivo2 Plus hypoxia workstation (Ruskinn, Leeds, UK) flooded with humidified gas mixture consisting of 5% CO_2_, 1% O_2_ and ~ 94% N_2_ at 37 °C. BEC were maintained for up to 12 h in glucose-free HBSS in the hypoxic chamber. Control cells were washed twice with HBSS containing 6 mM glucose equilibrated to standard oxygen levels. Control BEC exposed to HBSS containing glucose were placed into a normoxic incubator (5% CO_2_ at 37 °C) for a corresponding time.

#### Capillary electrophoresis

Whole cell proteins were isolated from either cultured BEC or freshly sorted BEC. Lysis buffer containing 25 mM Tris–HCl (pH 7.6), 150 mM NaCl, 1% Nonidet P-40, 1% sodium deoxycholate, 0.1% SDS and 1xHALT-Protease and Phosphatase Inhibitor Cocktail (Thermo Fisher Scientific, #78,440) was added to cell samples followed by an incubation on ice for 15 min. Cell lysates were transferred into centrifuge tubes, incubated on ice for additional 15 min and vortexed extensively at 5 min intervals. Cell homogenates were centrifuged for 15 min at 16,100xg at 4 °C. Supernatant was aliquoted and Bradford assay was used for protein concentration quantification. Protein expression was analyzed using a Wes instrument from ProteinSimple (San Jose, CA, USA) according to manufacturer's instructions. Samples were diluted with 0.1 × sample buffer (ProteinSimple, #042–195) to a working concentration of 0.15–0.5 µg/µl. Fluorescent 5 × master mix was added followed by an incubation at 95 °C for 5 min. The primary antibodies were diluted in Antibody diluent 2 (ProteinSimple, #042–203). Wes microplate was loaded with samples and reagents (biotinylated protein ladder, blocking reagent, primary antibodies, HRP-conjugated secondary antibody, streptavidin-HRP, luminol-peroxidase mix and washing buffer). All reagents were purchased from ProteinSimple, except primary antibodies. All antibodies are summarized in Table S3. Electrophoresis and chemiluminescence immunodetection were performed with default settings of the Wes instrument using a CCD camera. Compass software (ProteinSimple) and FIJI software were used for data analysis and visualization of chemiluminescence signal intensity values. Protein expression was calculated as integral of the chemiluminescence intensity (AUC).

#### Quantitative real-time RT-PCR analysis

RNA isolation from cells, cDNA synthesis, and quantitative real-time PCR were performed as described recently [12]. Primers were purchased from Eurofins Genomics (for primer sequences, see Table S5).

#### RNA sequencing

Total RNA from cultured BEC was purified using the Qiagen RNeasy Mini Kit (Qiagen, Hilden, Germany, #74,104) according to manufacturer's instructions. RNA quality was determined by Agilent BioAnalyzer 2100 (Agilent Technologies, Santa Clara, CA, USA). RNA samples had to meet the following requirements: RIN/RQN values ≥ 7.0 and 28S/18S ≥ 1.0. RNA sequencing analysis (strand specific mRNA sequencing on DNBSEQ platform, paired end 100 bp) and library preparation were performed by BGI Hong Kong. RNA data visualization, interpretation and analysis were performed with BGI’s Dr. Tom System. *Bowtie2* was applied to align the clean reads to the gene set. Gene expression levels were calculated by RSEM (v1.2.28) to get read count, FPKM and TPM. The analysis of differentially expressed genes (DEGs) with *q* < 0.05. was performed using the DESeq2 or DEGseq or PossionDis (for details, see: https://eu-biosys.bgi.com/help/en/mrna/).

#### Statistical analysis

Data plotting and statistical analyses were done in Prism 8 (GraphPad Software, La Jolla, CA, USA). If not indicated otherwise, all results are expressed as mean ± standard deviation (SD). Differences between two independent experimental groups were analyzed by two-tailed Student's *t* test (numerical data) or Mann–Whitney *U* rank-sum test (ordinal data). Differences in one parameter between three or more independent experimental groups were analyzed by using one-way ANOVA followed by a Holm-Sidak's multiple comparisons test. Differences in two parameters between two or more independent experimental groups were analyzed by two-way ANOVA followed by a Holm-Sidak's multiple comparisons test. A probability value of *P* < 0.05 was considered statistically significant.

## Results

### Ischemic conditions trigger Nfat5 expression in brain endothelial cells

**Fig. 1:**
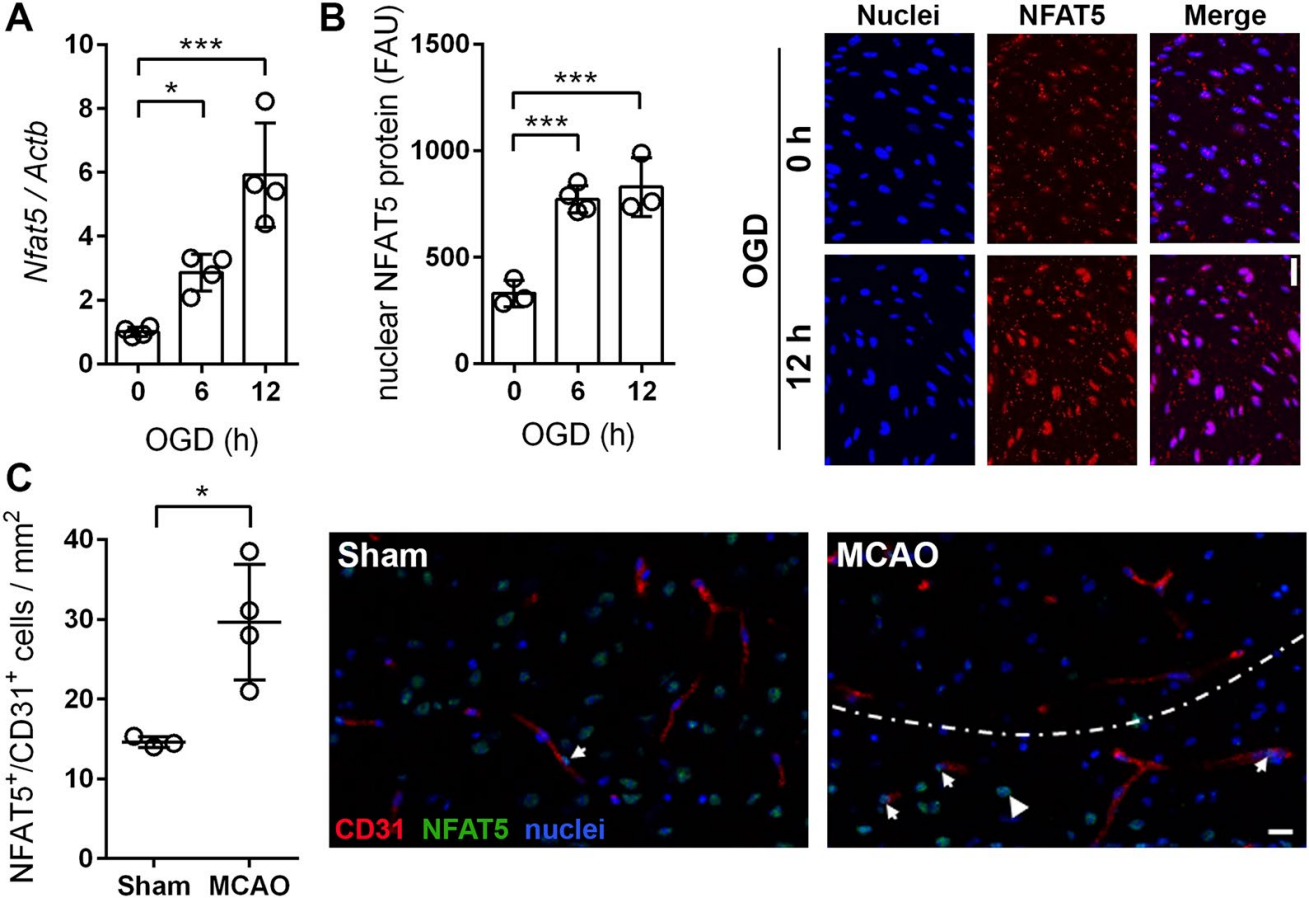
Ischemic stress increases the expression of NFAT5 in cerebral endothelial cells. Endothelial cells derived from microvessels prepared from brains of adult *Nfat5*^*fl/fl*^ mice were exposed to OGD conditions (glucose-free aCSF; 1% O_2_) for 6 or 12 h. Cells exposed to aCSF (+ Glc) under normoxic conditions for 12 h are used as control. **A** Real-time RT-PCR was applied to determine *Nfat5* transcript levels. Values are normalized to *Actb* and expressed as fold change of control (*n* = 4 per group; One-way ANOVA with Holm-Sidak's multiple comparisons test; * *p* < 0.05, *** *p* < 0.001). **B** Nuclear NFAT protein abundance was quantified by immunofluorescence staining (*n* = 3–4 per group; One-way ANOVA with Holm-Sidak's multiple comparisons test; *** *p* < 0.001). Representative microphotographs: NFAT5 (red), DAPI (blue). Scale bar: 50 µm.** C** *Nfat5*.^*fl/fl*^ mice underwent 45 min of MCAO followed by 24 h reperfusion. Sham-operated mice served as control. NFAT5 protein abundance in brain endothelial cells was determined by co-immunofluorescence staining (*n* = 3–4 per group; unpaired two-tailed Student's *t* test; * *p* < 0.05). Representative microphotographs: NFAT5 (green), CD31 (red), DAPI (blue). Arrows point at NFAT5 in nuclei of EC. The arrowhead tags a non-endothelial cell nucleus. The dotted line marks the border between ischemic and non-ischemic areas as evidenced by the lack of NFAT5 in the nuclei of non-endothelial cells (scale bar: 20 µm)

Brain endothelial cells (BEC) must rapidly adapt to changing environmental conditions in order to preserve a continuous distribution of oxygen and nutrients in the brain. Considering the general relevance of NFAT5 for controlling cellular responses to different types of environmental stressors, we assumed that ischemia promotes activation of this transcription factor in BEC. This hypothesis was initially tested by depriving cultured mouse BEC from oxygen and glucose (OGD) and assessing the expression as well as the accumulation of NFAT5 in the nucleus—a prerequisite for its transcriptional activity. As evidenced by qPCR and immunofluorescence-based analyses, ischemic conditions increased both the mRNA expression of *Nfat5* (Fig. 1A) and its nuclear accumulation (Fig. 1B) within 6 h. The relevance of this finding was examined in brains of mice subjected to acute I/R injury. In line with the in vitro results, the level of nuclear NFAT5 was significantly increased predominantly in BEC located in the peri-infarct zone adjacent to the ischemic core (Fig. 1C).

### Loss of Nfat5 in mouse BEC aggravates neuronal damage and functional deficiency after ischemic stroke

**Fig. 2:**
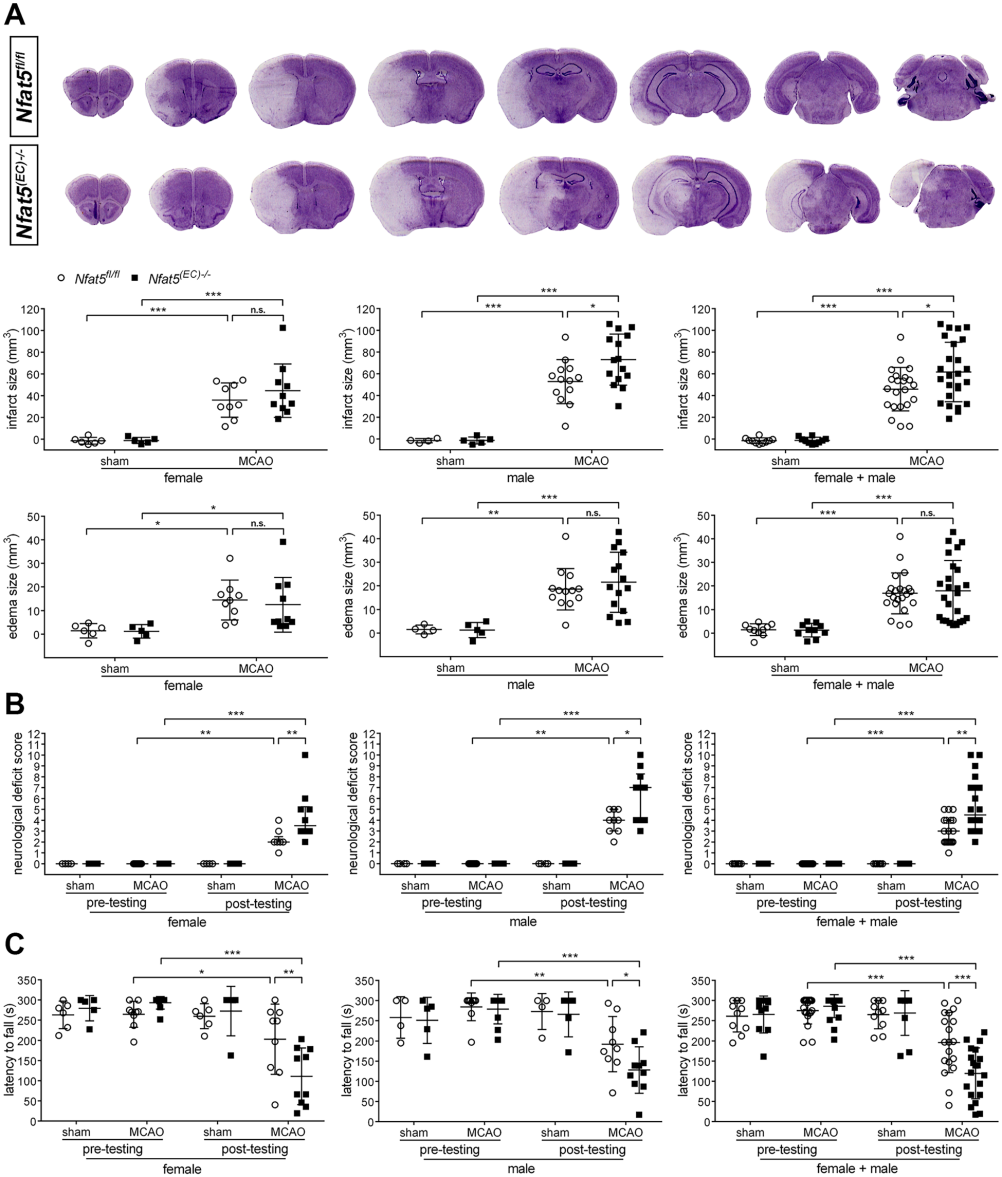
Endothelial cell-specific knockout of *Nfat5* worsens brain tissue damage and sensorimotor impairment in mice upon acute ischemic stroke. **A** *Nfat5*^*(EC)−/−*^ and *Nfat5*^*fl/fl*^ mice were subjected to 45 min of MCAO followed by 24 h reperfusion. Sham-operated mice served as control. Infarct and edema volume were determined by cresyl violet staining (*n* = 5–10 (♀)/*n* = 4–15 (♂) per group; Two-way ANOVA with Holm-Sidak's multiple comparisons test; * *p* < 0.05, ** *p* < 0.01, *** *p* < 0.001). **B** Neurological function was assessed using the modified neurological severity score (median with interquartile range; *n* = 5–10 (♀)/*n* = 4–10 (♂) per group; Wilcoxon matched-pairs signed-rank test (pre- vs post-MCAO), Mann–Whitney *U* rank-sum test (*Nfat5*^*(EC)−/−*^ vs *Nfat5*.^*fl/fl*^); * *p* < 0.05, ** *p* < 0.01, *** *p* < 0.001). **C** Motor function was evaluated by using the Rotarod performance test (*n* = 5–10 (♀)/*n* = 4–10 (♂) per group; Two-way ANOVA with Holm-Sidak's multiple comparisons test; * *p* < 0.05, ** *p* < 0.01, *** *p* < 0.001)

The aforementioned results prompted us to study the impact of NFAT5-mediated transcription in BEC on the outcome of ischemic stroke. To this end, we generated endothelial cell-specific *Nfat5* gene knock-out mice (*Nfat5*^*(EC)−/−*^) by crossing mice harboring two floxed alleles of *Nfat5* with transgenic mice expressing CreERT2 recombinase under control of the *vascular endothelial cadherin* (*VE-cadherin*, also known as *cadherin 5* (*Cdh5*)) gene promoter followed by CreERT2 activation through application of tamoxifen followed by a recovery period of three weeks. Littermate mice receiving miglyol as solvent control were used as controls (*Nfat5*^*fl/fl*^). In BEC separated from brain single cell suspensions of *Nfat5*^*(EC)−/−*^ mice, *Nfat5* transcript levels were reduced by 80% as compared to those from *Nfat5*^*fl/fl*^ mice confirming successful genetic ablation of *Nfat5* (Supplement S1). The cross-cohort analysis of mice subjected to experimental ischemic stroke revealed a significantly increased infarct lesion size in *Nfat5*^*(EC)−/−*^ mice as compared to *Nfat5*^*fl/fl*^ littermate mice (Fig. 2A). This was, however, much more pronounced in male as compared to female *Nfat5*^*(EC)−/−*^ mice reflecting gender-dependent responses, which have been reported for this model [6]. In contrast, stroke-associated edema formation did not differ markedly between *Nfat5*^*(EC)−/−*^ and *Nfat5*^*fl/fl*^ mice, regardless of gender (Fig. 2A). Importantly, aggravated cerebral tissue damage was accompanied by a significantly increased sensorimotor impairment of female and male *Nfat5*^*(EC)−/−*^ mice in comparison to sex-matched controls (Fig. 2B, C).

Notably, the increased infarct volume detected in *Nfat5*^*(EC)−/−*^ mice 24 h upon onset of stroke was not accompanied by an altered degree of apoptosis (**Supplement S3A**) or degenerating neurons (Supplement S3) across the ipsilateral striatum (infarct core) and cortex (ischemic peri-infarct area; specification of anatomical sub regions is shown in Supplement S2). Thus, *Nfat5* deficiency in BEC may not substantially affect the overall cell death in the late acute phase after stroke.

**Fig. 3:**
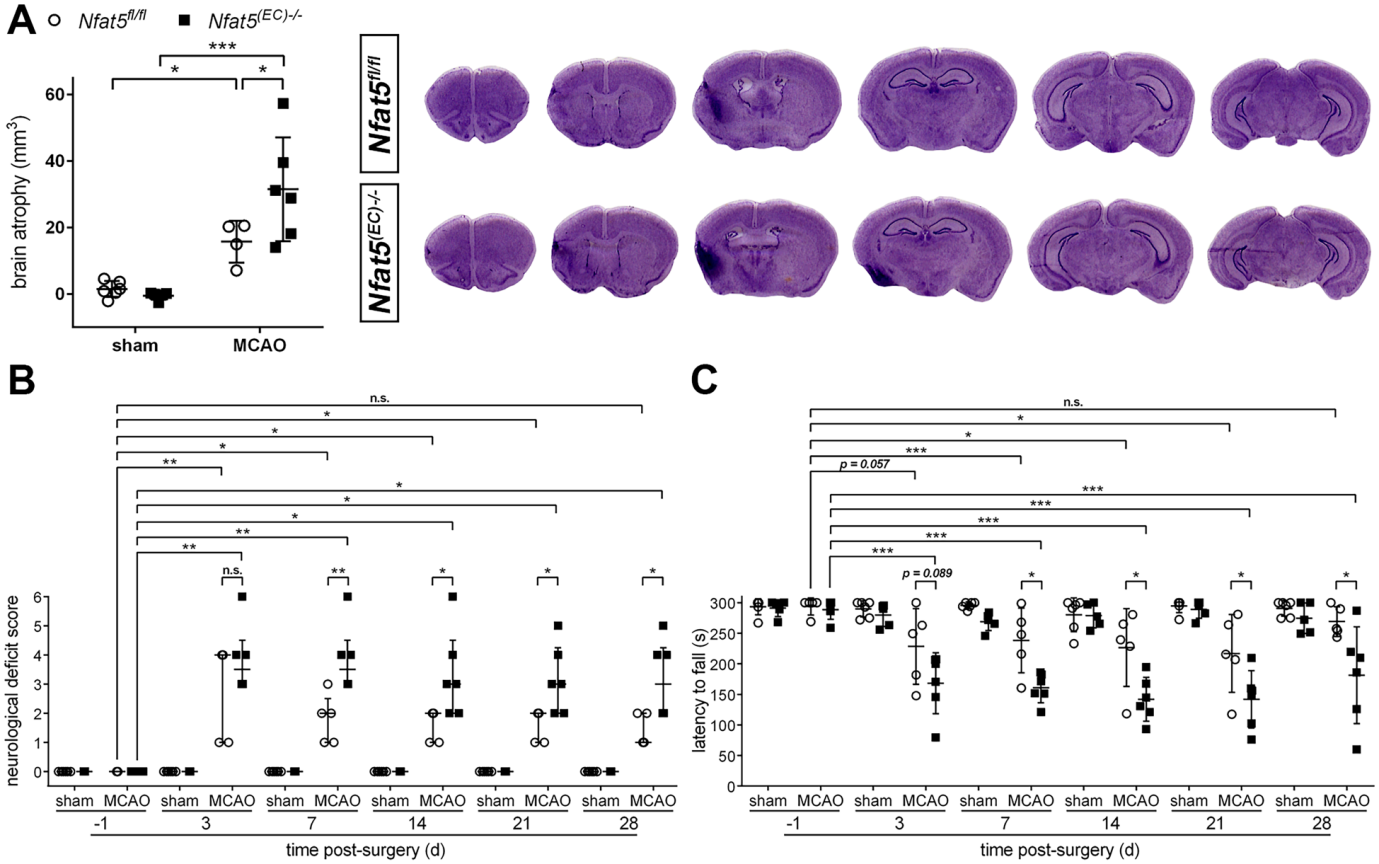
Endothelial cell-specific knockout of *Nfat5* worsens brain tissue loss and sensorimotor impairment in mice during the chronic phase of ischemic stroke. **A** *Nfat5*^*(EC)−/−*^ and *Nfat5*^*fl/fl*^ mice were subjected to 45 min of MCAO followed by 28 d reperfusion. Sham-operated mice served as control. Cerebral atrophy was assessed by morphovolumetric analysis of cresyl violet-stained brain tissue sections (*n* = 5–6 per group; Two-way ANOVA with Holm-Sidak's multiple comparisons test; * *p* < 0.05, *** *p* < 0.001). **B** Neurological function was assessed using the modified neurological severity score (median with interquartile range; *n* = 5–6 per group; Kruskal–Wallis test with Dunn's multiple comparisons test (pre- vs post-MCAO), Mann–Whitney *U* rank-sum test (*Nfat5*^*(EC)−/−*^ vs *Nfat5*^*fl/fl*^); * *p* < 0.05, ** *p* < 0.01). **(C)** Motor function was evaluated by using the Rotarod performance test (*n* = 5–6 per group; One-way repeated measures ANOVA with Holm-Sidak's multiple comparisons test (pre- vs post-MCAO), unpaired two-tailed Student's *t* test (*Nfat5*^*(EC)−/−*^ vs *Nfat5*.^*fl/fl*^); * *p* < 0.05, *** *p* < 0.001)

We next monitored the histopathological and functional outcome during the chronic phase after ischemic stroke. Evaluation of brain atrophy, whose extent is predominantly determined by brain tissue damage occurring in the acute phase, four weeks upon onset of stroke revealed significantly reduced loss of brain tissue in *Nfat5*^*(EC)−/−*^ mice as compared to *Nfat5*^*fl/fl*^ animals (Fig. 3A). Accordingly, the general neurological status (Fig. 3B) as well as the motor performance (Fig. 3C) of *Nfat5*^*(EC)−/−*^ mice were much more aggravated throughout the entire observational period in comparison to *Nfat5*^*fl/fl*^ littermates.

### Integrity of the brain endothelium and its proliferation are not affected by knockout of Nfat5

Considering the relevance of brain blood vessels for tissue (re)perfusion, we assumed that the vascular integrity or response may be impaired in brain of *Nfat5*^*(EC)−/−*^ versus *Nfat5*^*fl/fl*^ mice, which may limit neuronal recovery after ischemia. In mice subjected to 45 min MCAO followed by 24 h of reperfusion, the density of both total and perfused vessel segments was significantly reduced within the striatum and to a lesser extent in the adjacent cortex (Supplement S4A, ). However, the decline of cerebral microvessels due to stroke was not different in *Nfat5*^*(EC)−/−*^ versus *Nfat5*^*fl/fl*^ mice (Supplement S4A, B). Moreover, the pericyte coverage of remaining microvessels, crucially important for maintenance of the BBB integrity, was comparable in the ipsilateral brain hemispheres of *Nfat5*^*(EC)−/−*^ and *Nfat5*^*fl/fl*^ mice subjected to either MCAO or sham surgery (**Supplement S4C**). Considering the comparable vasogenic edema formation in *Nfat5*^*fl/fl*^ and *Nfat5*^*(EC)−/−*^ mice mentioned above (Fig. 2A), our results do not point to a major role of endothelial NFAT5 for maintenance of the cerebrovascular integrity during ischemic stroke.

Adaptive angiogenic responses in vascular endothelial cells contribute to brain tissue repair and functional recovery in the sub-acute and chronic phases following stroke. Angiogenesis, which mainly refers to the sprouting of new vessels from pre-existing capillaries, requires a concerted migration and proliferation of endothelial cells. For pulse labeling of actively proliferating cells, BrdU has been repeatedly applied to mice between day 3 and 6 after onset of stroke. One month upon stroke, the number of BrdU/CD31 double positive cells was significantly increased in the ipsilateral hemisphere of mice subjected to MCAO as compared to sham-operated animals, whereas proliferating endothelial cells were predominantly localized within the striatum (Supplement S5A). However, frequency and spatial distribution of proliferating endothelial cells were not significantly different between *Nfat5*^*fl/fl*^ and *Nfat5*^*(EC)−/−*^ mice (Supplement S5A). Accordingly, the density of total and perfused microvessels across striatum, cortex or entire ipsilateral brain hemisphere was comparable in both experimental groups (Supplement S5B, C). These observations suggest that endothelial NFAT5 is rather neglectable for the compensatory neovascularization in response to cerebral ischemia.

Immediately after onset of cerebral ischemia, injured and dying neurons release damage-associated molecular patterns (DAMPs) which promotes local pro-inflammatory activation of brain-resident cells, including microglia, astrocytes, and endothelial cells triggering recruitment and infiltration of peripheral leukocytes. Local brain inflammation, resulting from neuronal damage in stroke, can aggravate a secondary injury and causes persisting global brain inflammation. Considering the relevance of NFAT5 for controlling immune responses [14], we determined quantity and phenotype of immune cells in single cell suspensions generated from mouse brains. Detailed flow cytometric analyses revealed that acute ischemic stroke increased the abundance of monocytes, macrophages, neutrophils and T cells in the brain (Supplement S6). However, the frequency of all studied leukocyte populations as well as the number of brain-resident microglia across the ipsilesional brain parenchyma was not significantly different between *Nfat5*^*(EC)−/−*^ and *Nfat5*^*fl/fl*^ mice (Supplement S6). Moreover, within the microglia and macrophage cell subsets the proportion of cells with a pro-inflammatory M1 phenotype (CD80^high^, MHCII^high^) and anti-inflammatory M2 phenotype (CD206^high^, MHCII^low^) was quite similar in both genotypes (Supplement S6).

### Nfat5 deficiency limits the expression of solute carriers and Kcnj2 in mouse BEC exposed to ischemic conditions

Collectively, loss of endothelial *Nfat5* resulted in expanded infarct sizes after brain I/R without specifically affecting vascular integrity, angiogenic activation or stroke-associated neuroinflammation. Moreover, a NFAT5-dependent neuroprotective mechanism originating from the BEC population appears unlikely given the unchanged degree of neuronal cell death in the late acute phase upon stroke.

**Fig. 4:**
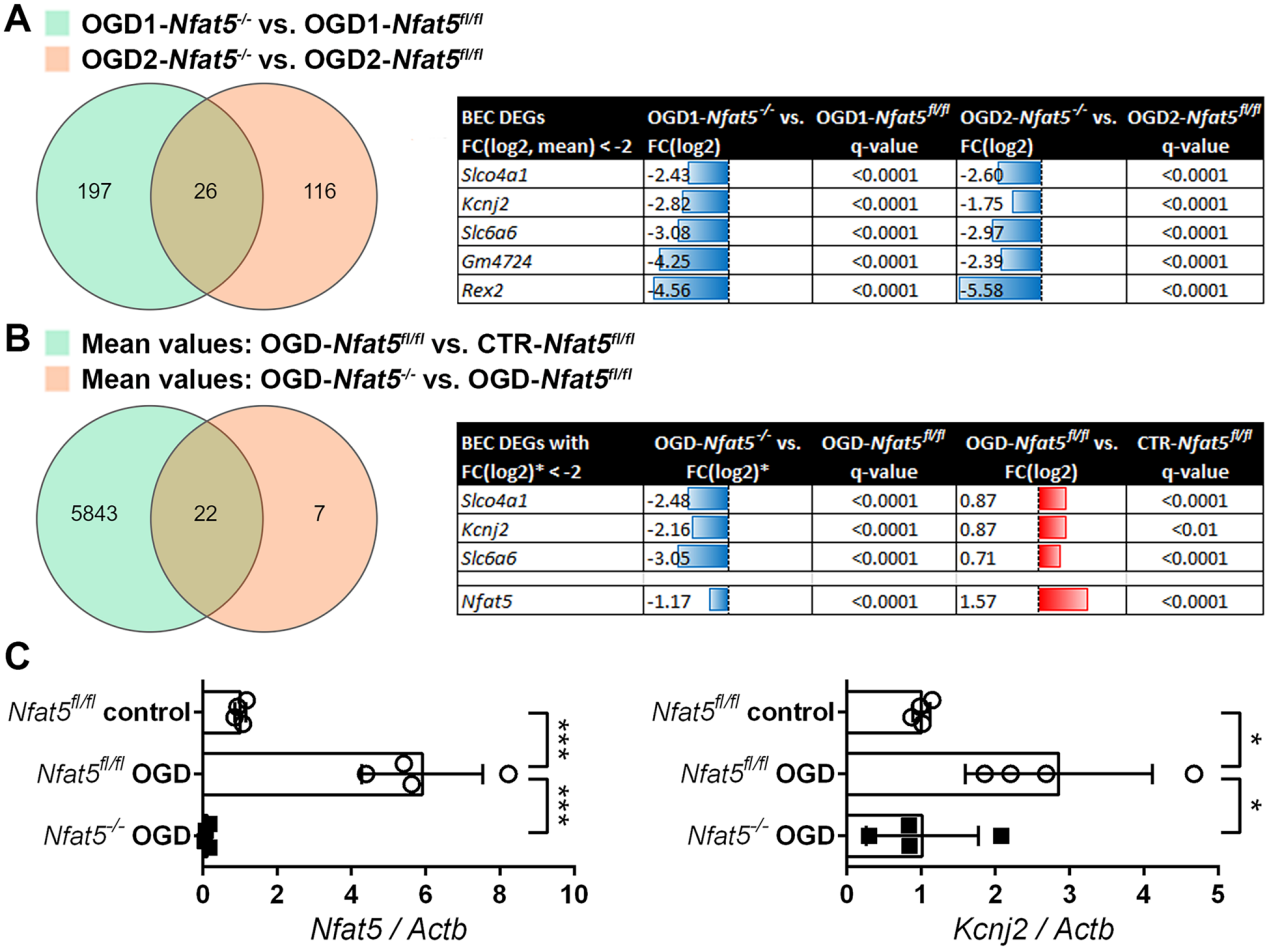
*Nfat5* knockout decreases expression of *Kcnj2* in OGD-exposed BEC. Primary cultures of *Nfat5*^*fl/fl*^ BEC were exposed to 1 µM 4-hydroxytamoxifen (*Nfat5*^*−/−*^) or solvent for 3 d followed by a recovery period of 3 d. BEC were exposed to OGD conditions (glucose-free aCSF; 1% O_2_) for 12 h. Cells exposed to aCSF (+ Glc) under normoxic conditions for 12 h are used as control (CTR). RNA isolated from BEC of two experimental approaches (performed in duplicate) was subjected to RNAseq analysis. **A** Venn diagram-based analysis to identify NFAT5-dependent differentially expressed genes (DEGs) in OGD-exposed BEC (located in the intersection). The table lists the DEGs meeting our selection criteria (fold change—FC(log2) < − 2 in at least one approach). **B** Venn diagram-based analysis to identify NFAT5-dependent differentially expressed genes (DEGs) in OGD-exposed BEC, which were also significantly upregulated in OGD-*Nfat5*^*fl/fl*^ versus CTR-*Nfat5*^*fl/fl*^ BEC (located in the intersection). The table lists the DEGs meeting our selection criteria (mean FC(log2) of all experimental approaches (OGD-*Nfat5*^*−/−*^ versus OGD-*Nfat5*^*fl/fl*^) < − 2 and mean FC(log2) (OGD-*Nfat5*^*fl/fl*^ versus CTR-*Nfat5*^*fl/fl*^) > 0.5). *Nfat5* expression is shown as reference. **(C)** Control of the expression of *Nfat5* and *Kcnj2* in BEC by real-time RT-PCR. Values are normalized to *Actb* and expressed as fold change of *Nfat5*.^*fl/fl*^ control (*n* = 4 per group; One-way ANOVA with Holm-Sidak's multiple comparisons test; * *p* < 0.05; *** *p* < 0.001)

To unravel the molecular origin of the pathomechanism explaining the enlarged infarct size, the histological changes, and functional deficits observed in *Nfat5*^*(EC)−/−*^ mice, we cultured EC isolated from mouse brains (Supplement S7), exposed them to control or OGD conditions for 12 h and isolated RNA from two individual experimental approaches (each in duplicates) to perform RNA sequencing (RNA-seq) analysis. The corresponding data were processed to identify differentially expressed genes (DEGs), whose expression were comparably regulated in both experimental approaches and affected by two distinct variables: (i) loss of *Nfat5* under OGD conditions (Fig. 4A) and (ii) OGD versus control conditions in *Nfat5*^*fl/fl*^ BEC (Fig. 4B). The corresponding Venn diagram-based comparative analyses identified three genes, which matched these cut-off criteria. The corresponding DEGs encoded a solute carrier organic anion transporter (*Slco4a1*), a sodium- and chloride-dependent taurine transporter (*Slc6a6*) and the inward rectifying potassium channel Kir2.1 (*Kcnj2*). While both *Slco4a1* and *Slc6a6* are known targets of NFAT5 [17, 18], the latter may in fact influence the outcome of ischemic stroke by regulating the transport of the neuroprotective endogenous amino acid taurine [35] through the BBB. However, ELISA-based analysis of brain tissue did not indicate a difference in the taurine content in *Nfat5*^*(EC)−/−*^ versus *Nfat5*^*fl/fl*^ mice (Supplement S8).

Interestingly, Kir2.1 has been shown to affect cerebral blood flow [10, 32] and may potentiate the activity of endothelial nitric oxide synthase (eNOS) [13]. Moreover, endothelial Kir2.1 function has been associated with vasodilation of brain blood vessels [46] and capillary-to-arteriole electrical signaling, contributing to cerebral blood flow.

### Loss of endothelial Nfat5 diminishes the level of Kir2.1 in arterial BEC

**Fig. 5:**
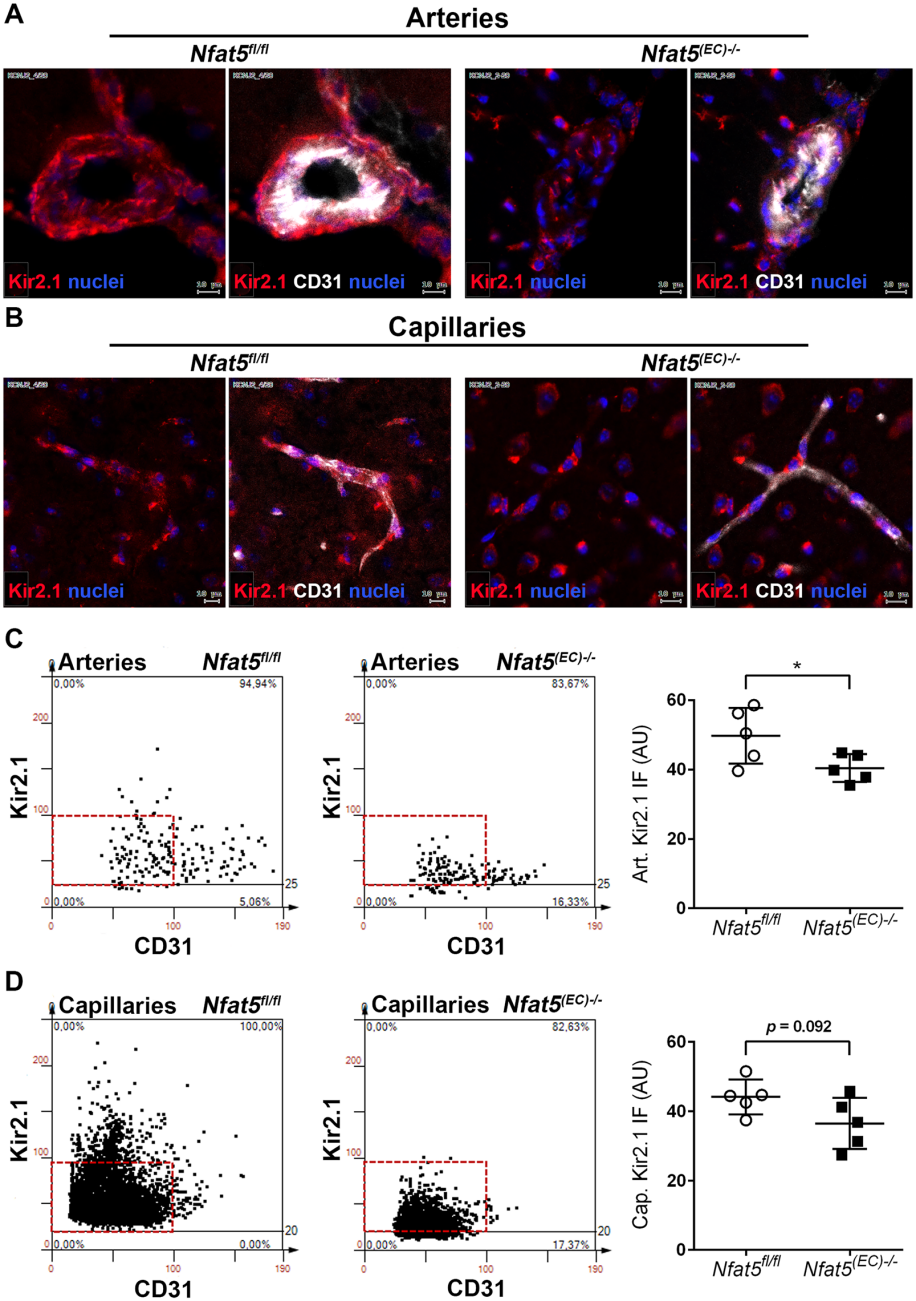
Automated immunofluorescence analysis of Kir2.1 (in vivo*).* Immunofluorescence-based detection of the endothelial cell marker PECAM1 (CD31) and Kir2.1 in brain arteries **A** and capillaries **B** of *Nfat5*^*fl/fl*^ and *Nfat5*^*(EC)−/−*^ mice (sham treatment). Images were processed and automatically analyzed by TissueFAXS (TissuGnostics, see Supplement S11 and S12 for details). Detection of CD31^high^ arterial and capillary structures in defined regions of interest (ROI) is followed by assessment of the corresponding Kir2.1-associated fluorescence. The data of representative ROIs (**C**: artery, **D**: capillaries) are plotted as scattergram (y-axis: Kir2.1 immunofluorescence level, x-axis: CD31 level). The mean Kir2.1 fluorescence values determined in all corresponding ROIs of individual mouse brains are summarized as bar graphs (n = 5 per group, unpaired two-tailed Student's *t* test (*Nfat5*^*(EC)−/−*^ vs *Nfat5*.^*fl/fl*^); * *p* < 0.05)

The RNAseq-based results spurred us to scrutinize the putative link between *Nfat5* and *Kcnj2* expression. To this end, we performed multiple in silico analyses, which indicated at least two highly probable NFAT5 binding sites in the promoter of the *Kcnj2* gene (Supplement S9). In fact, the protein level of Kir2.1 decreased in cultured mouse brain endothelial cells upon knockout of *Nfat5* (Supplement S10). Moreover, meta-analyses of two separate single cell-RNAseq data sets from mouse brain cells published by [47] and [21] suggested that both *Nfat5* and *Kcnj2* are most strongly expressed in arterial endothelial cells (Supplement S11A-C). Similarly, we detected NFAT5 preferentially in nuclei of arterial EC of *Nfat5*^*fl/fl*^ brains by applying immunofluorescence-based analyses (Supplement S12A). Likewise, the Kir2.1-associated fluorescence signal was more intense in arterial versus capillary BEC (Supplement S12B). Moreover, exemplary analysis of RNA extracted from BEC isolated from *Nfat5*^*fl/fl*^ and *Nfat5*^*(EC)−/−*^ brains showed a decrease of *Kcnj2* expression in *Nfat5*-deficient BEC (Supplement S12C). These findings suggest that the Kir2.1 level might be regulated in a NFAT5-dependent and BEC subtype-specific manner, which is ischemia-independent. We therefore performed an automated immunofluorescence/TissueFAXS-based analysis of brain hemispheres. To this end, capillaries close to the expected infarct area as well as (alpha smooth muscle actin, aSMA-positive) branches of the MCA were subjected to an automated image analysis determining the intensity of the Kir2.1-specific immunofluorescence signal in CD31-positive EC (Supplement S13). We observed a slightly elevated Kir2.1-specific fluorescence signal intensity in *Nfat5*^*fl/fl*^ arterial versus capillary BEC that significantly increased in arterial but not capillary BEC after MCAO (Supplement S14). Most importantly, corresponding results revealed lower Kir2.1 levels in arteries but not capillaries of (sham) *Nfat5*^*fl/fl*^ versus *Nfat5*^*(EC)−/−*^ mice—independent from I/R conditions (Fig. 5).Endothelial Kir2.1 in brain arteries or capillaries of *Nfat5*^*(EC)−/−*^ mice appeared not much regulated within 24 h after MCAO (Supplement S15). However, we partially observed higher Kir2.1 levels in arterial endothelial cells after MCAO (Supplement S15A) suggesting their capacity to overcome an initial depletion of Kir2.1 in principle.

### Endothelial Nfat5 deficiency limits the reperfusion capacity after ischemic stroke

**Fig. 6:**
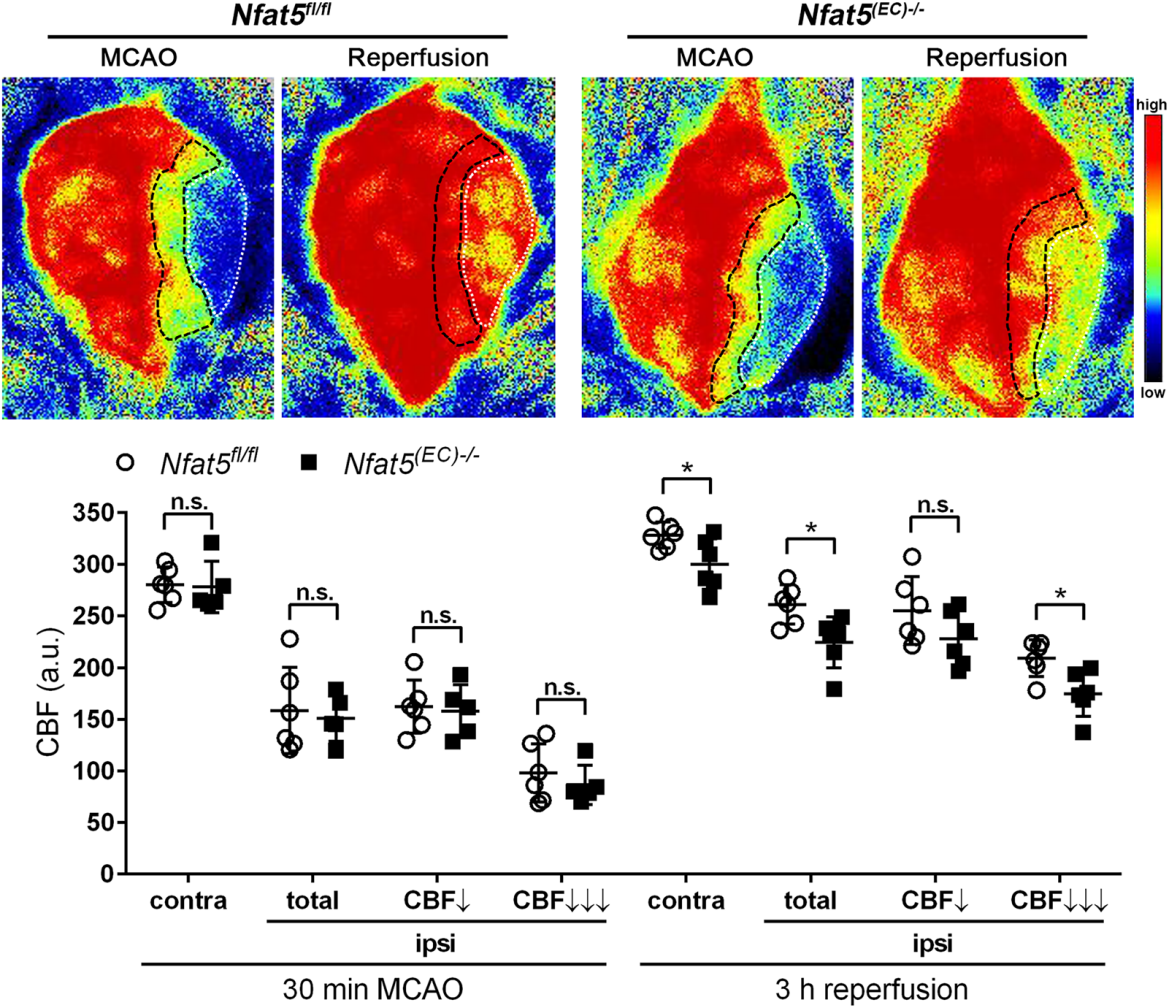
Endothelial cell-specific knockout of *Nfat5* critically affects the reperfusion of the MCA-supplied brain territory. *Nfat5*^*(EC)−/−*^ and *Nfat5*.^*fl/fl*^ mice were subjected to 45 min of MCAO followed by 3 h reperfusion. Cortical blood flow (CBF) was measured on the skull of living mice using high-resolution laser speckle contrast imaging (LSCI). Representative LSCI perfusion images of the cortical surface at 30 min MCAO and 3 h upon reperfusion. Color bar code: The colors from blue to red represent the blood flow velocity from lower to higher. CBF is given in arbitrary units (a.u.). ROIs across the ipsilateral cerebral cortex were defined according the following CBF thresholds: strongly hypoperfused area (CBF < 40% of mean contralateral cerebral cortex during MCAO, CBF↓↓↓), moderately hypoperfused region (CBF between 40 and 70% of mean contralateral cerebral cortex during MCAO, CBF↓) (*n* = 6 per group; unpaired two-tailed Student's *t* test; * *p* < 0.05)

Thus far, our results suggested that NFAT5 maintains the baseline Kir2.1 level prior ischemia or reperfusion. Considering the reported impact of Kir2.1 on vasorelaxation [10, 32], we investigated the functional relevance of the aforementioned findings by comparing the compensatory post-occlusive hyperemia of the ipsilateral MCA area in *Nfat5*^*fl/fl*^ and *Nfat5*^*(EC)−/−*^ mice. High-resolution laser speckle contrast imaging (LSCI) confirmed a substantial reduction of cortical cerebral blood flow by − 40 to − 60% upon MCAO (Fig. 6). However, cortical blood flow in both contralateral and ipsilateral cortices during MCAO was quite similar in *Nfat5*^*fl/fl*^ and *Nfat5*^*(EC)−/−*^ mice (Fig. 6). MCA re-opening provoked a gradual reperfusion of both moderately and strongly hypoperfused cortical areas, albeit not completely when compared to the contralateral cortical blood flow 3 h after onset of reperfusion (Fig. 6). The reperfusion of the entire ipsilateral cortex and even more of the strongly hypoperfused cortical region was significantly less pronounced in *Nfat5*^*(EC)−/−*^ mice as compared to *Nfat5*^*fl/fl*^ littermates (Fig. 6). Interestingly, restoration of blood flow through the ipsilesional MCA (left cerebral cortex) also caused an elevated perfusion of the non-affected right cerebral cortex of *Nfat5*^*fl/fl*^ mice. By contrast, no post-occlusive hyperperfusion of the structurally intact right cerebral cortex in *Nfat5*^*(EC)−/−*^ mice was detected (Fig. 6).

Collectively, our data indicate that NFAT5 is required to maintain the baseline expression of *Kcnj2* preferentially in arterial endothelial cells of the cerebral vasculature. This appears to be a prerequisite for adequate post-occlusive reperfusion of the infarct area limiting the expansion of the infarct area and the degree of functional impairment.

## Discussion

Timely recanalization of occluded brain arteries by intravenous thrombolysis and/or endovascular thrombectomy is currently the first choice for acute ischemic stroke management and has effectively improved the clinical outcome of stroke patients [20]. In fact, corresponding therapeutic interventions reflect the importance of a swift (re)initiation of brain perfusion as the most relevant determinant to limit the infarct size and consequently the degree of functional failure [15]. Accordingly, the phrase “time is brain” was created and underscored by introducing appropriate formula for calculating the rate of tissue loss. It was estimated that an average stroke patient loses 1.9 million neurons, 13.8 billion synapses, and 12 km of axonal fibres every minute if left untreated [41].

However, it is worth noting that successful recanalization might not be equivalent to successful reperfusion as recent clinical studies have demonstrated that a number of patients still suffered from sustained hypoperfusion in certain areas despite successful recanalization and the full patency of the occluded artery being achieved [20]. As such, the deficiency of perfusion after successful recanalization is known as the “no-reflow phenomenon” (NRP). NRP severely affects functional recovery of stroke patients, and is associated with functional and structural alterations of the cerebrovascular microcirculation [20, 45]. Even though the underlying mechanisms are not fully understood, NRP was reported to result from mechanical obstruction by blood components (e.g. platelets, leukocytes, and fibrin) or clot fragmentation, and compression due to functional or structural change in the vessel wall or the surrounding cells [20, 45]. The latter primarily comprises: (i) excessive pericyte contraction, (ii) increased vasoconstriction of parenchymal arterioles, and (iii) swelling (edema) of endothelial cells and perivascular astrocyte end-feet [20, 45].

Our findings delineated a NRP based on an inadequate or delayed blood flow recovery after acute ischemic stroke in mice, upon loss of endothelial NFAT5. While *Nfat5* deficiency evoked more pronounced infarct sizes and neurological deficit scores in male mice as compared to females, males also showed an overall stronger basic response to the MCAO than females. This phenomenon has been repeatedly reported for the MCAO model and is most likely caused by an estrogen-dependent protective in part reperfusion-preserving mechanism [2, 11, 39, 55]. Consequently, despite being caused by a comparable effect strength, absolute values of infarct sizes and neurological deficit scores will be lower in *Nfat5*-deficient female versus male mice as they originate from less pronounced baseline effects.

Although the overall protective impact of endothelial NFAT5 on the stroke severity may clearly be deduced from our data, the delineation of the origin of the observed effects was challenging. Considering that loss of endothelial NFAT5 did neither affect edema formation, inflammation and angiogenesis nor did this further alter structural and functional parameters at later stages after stroke such as brain atrophy, we concluded that NFAT5 acts in the immediate early phase after MCAO. In fact, expansion of the infarct area in *Nfat5*^*(EC)−/−*^ versus *Nfat5*^*fl/fl*^ mice within 24 h after onset of reperfusion without alteration of the degree of dying neurons appeared to be the most substantial observation. As the infarct size is greatly influenced by the level of reperfusion after MCAO, impairment in perfusion recovery as observed in *Nfat5*^*(EC)−/−*^ versus *Nfat5*^*fl/fl*^ mice is a plausible explanation for the exacerbated consequences of brain ischemia.

With respect to the mechanism by which endothelial NFAT5 supports reperfusion, we did not find any evidence that its genetic ablation impairs the expression of vasodilation-associated genes such as cyclooxygenase or nitric oxide synthase (data not shown). In general, NFAT5 has been reported to modulate gene expression by (i) directly stimulating the expression of target genes [7], (ii) enhancing gene expression under the control of other transcription factors [29, 33] and (iii) epigenetic suppression of gene expression [28, 30]—dependent on the environmental stressor and cell type. However, our in vitro findings indicated that NFAT5 takes six hours under ischemic conditions to enter the nucleus as a prerequisite to exert its activity. Similar observations were made in cells exposed to biomechanical stimulation [53]. Given the fact that the deficit in reperfusion was assessable within the first three hours after MCAO, it is unlikely that it was caused by a direct ischemia-induced and NFAT5-mediated change in transcription. Instead, our results imply that NFAT5 preferentially accumulates in nuclei of arterial endothelial cells to maintain BEC gene expression in homeostasis. We recently reported that the mice used in this study do not develop any obvious phenotype after genetic ablation of *Nfat5* in endothelial cells under control conditions [27]. Its relevance became nevertheless overt upon environmental conditions requiring a transcriptional adaptation such as hypoxia. In the context of brain ischemia, the transcriptional regulation of gene expression by NFAT5 appears to be similarly relevant for adequate stress responses of the vasculature. However, this obviously depends on the EC subtype-specific levelling of gene expression preceding the reperfusion following an artery occlusion.

On the molecular level, knockout of *Nfat5* resulted in a decrease in the level of the inwardly rectifying potassium channel Kir2.1, which was most pronounced in arterial BEC. In line with this, in silico analyses revealed two putative NFAT5 binding sites within the promoter of the Kir2.1 encoding gene *Kcnj2*. While we cannot exclude dysregulation of other NFAT5-controlled vasoregulatory gene products in BEC under the chosen experimental conditions, a depletion of Kir2.1 in arterial BEC provides a plausible explanation for a deficit in vascular relaxation after ischemic conditions. It has been previously shown that Kir2.1 activity in BEC is of utmost importance for the local neuronal activity-dependent increase in cortical cerebral blood flow (functional hyperemia). Kir2.1 channels are activated by extracellular K^+^, which accumulates during neural activity to produce a rapidly propagating retrograde hyperpolarization that may spread over vascular smooth muscle cells via myoendothelial gap junctions [19] to cause upstream arteriolar dilation, increasing blood flow into the capillary bed [10, 32]. In line with these observations, targeted ablation of *Kcnj2* in mice inhibits cerebral artery dilation [54], which was however originally attributed to the effect on vascular smooth muscle cells—a theory that has been challenged meanwhile [13]. In fact, endothelial Kir2.1 was shown to mediate vasodilation of murine arteries in a potassium-dependent manner, which was abolished upon knockout of endothelial Kir2.1 [43]. Likewise, loss of Kir2.1 was shown to interfere with the flow-induced eNOS-dependent generation of NO [1]. Finally, in mouse models of cerebral small vessel disease, Alzheimer's Disease, and traumatic brain injury impaired functional hyperemia has been attributed to reduced activity of capillary Kir2.1 channels [10, 37, 38].

Collectively, our work revealed a critical role of endothelial *Nfat5* for limiting infarct development after brain ischemia underlining its relevance in the context of organotypic stress responses. Our findings imply that loss of endothelial *Nfat5* causes a reperfusion deficit after brain ischemia most likely by a general depletion of Kir2.1 in the brain endothelium.

## Supplementary Information

Supplementary file 1.

## Data Availability

The data underlying this article are available in the article and in its Supplementary material online. Additional datasets used and/or analysed during the current study will be made available from the corresponding author on reasonable request.
