# Supplementary material for: Adequate post-ischemic reperfusion of the mouse brain requires endothelial NFAT5
Source: Acta Neuropathol Commun. 2024 Dec 22;12:200. doi: 10.1186/s40478-024-01918-5 (PMC11663326; doi:10.1186/s40478-024-01918-5)
Supplement: Supplementary file 1 [file 40478_2024_1918_MOESM1_ESM.pdf]

SUPPLEMENTARY DATA

Adequate post-ischemic reperfusion of the mouse brain requires endothelial NFAT5

Reiner Kunze [1,†], Paul Wacker [1,†], Paula Breuer [1], Emil Nasyrov [1,2], Ivan M. Kur [3], Andreas Weigert [3], Andreas H. Wagner [1], Hugo H. Marti [1], Thomas Korff [1,4,*]

[1] Institute of Physiology and Pathophysiology, Department of Cardiovascular Physiology, Heidelberg University, Heidelberg, Germany

[2] Centre for Ophthalmology, University Eye Hospital Tuebingen, Tuebingen, Germany

[3] Institute of Biochemistry I, Faculty of Medicine, Goethe University Frankfurt, 60590 Frankfurt am Main, Germany

[4] European Center for Angioscience (ECAS), Medical Faculty Mannheim, Heidelberg University, 69120 Heidelberg, Germany

* Correspondence: korff@physiologie.uni-heidelberg.de

† These authors contributed equally to this work.

Table S1 List of primers used to genotype mice

Allele	Primer sequence (5´-3´)	Size (bp) of amplicon
Cdh5-cre/ERT2	Fwd GGCGCGGCAACACCATTTTT	wt: -
	Rev CCGGGCTGCCACGACCAA	tg: 445
Nfat5 floxed	Fwd GTAACCATGATTAGTCTTTTAGCTTTATG	wt: 334
	Rev GTTCTGAGAATCCAAAGCACAAC	floxed: 392

Table S2 Overview of animals that met defined exclusion criteria

Animals that met the following criteria were excluded from end-point analyses: (1) death during surgery due to procedural or anesthetic problems, (2) death before sampling, (3) weight loss >20% post-surgery, (4) intracerebral hemorrhage, (5) structural brain abnormalities (e.g. hydrocephalus), and (6) no infarction (only MCAO).

mouse line/strain		number of mice met exclusion criteria (% of total animals)						
		(1)	(2)	(3)	(4)	(5)	(6)	total
Nfat5$^{fl/fl}$	sham	–	–	–	–	–	X	0 / 19 (0 %)
	MCAO	–	–	1	–	–	–	1 / 51 (2 %)
Nfat5$^{(EC)-/-}$	Sham	–	–	–	–	–	X	0 / 20 (0 %)
	MCAO	–	1	–	–	–	–	1 / 52 (2 %)

Table S3 List of antibodies used for capillary electrophoresis (c. e.) and immunofluorescence (IF) staining

Antibody type	Immunogen	Host species	Clonality	Conjugate	Supplier	Catalog no.	Dilution	Application
Primary	ms CD31	rt	monoclonal	/	BD Biosciences	553370	1:100	IF (tissue)
Primary	ms CD31	rt	monoclonal	/	BD Biosciences	550274	1:10	IF (tissue)
Primary	ms CD31	gt	polyclonal	/	R&D systems	AF3628	1:200	IF (cells)
Primary	BrdU	sh	polyclonal	/	Novus Biologicals	500-235	1:200	IF (tissue)
Primary	ms PDGFRβ	rb	monoclonal	/	Cell Signaling Technology	3169	1:100	IF (tissue)
Primary	ms NFAT5	ms	monoclonal	/	Santa Cruz Biotechnology	sc-398171	1:30	c. e.
Primary	ms NFAT5	rb	polyclonal	/	Novus Biologicals	NB120-3446	1:50	IF (tissue)
Primary	ms NFAT5	rb	polyclonal	/	Invitrogen	PA1-023	1:200	IF (cells)
Primary	ms KCNJ2	rb	polyclonal	/	LifeSpan BioScienes	LS-B15876	1:200	IF (tissue)
Primary	ms αSMA	ms	polyclonal	FITC	Sigma	F3777	1:200	IF (tissue)
Primary	hu NOS3	gt	polyclonal	/	R&D systems	AF950	1:20	c. e.
Primary	ms TUBA1A	rb	polyclonal	/	Cell Signaling Technology	CS#2144	1:20	c. e.
Primary	ms ACTB	ms	monoclonal	/	R&D systems	MAB8929	1:800	c. e.
Secondary	gt IgG	dk	polyclonal	HRP	R&D systems	HAF109	1:50	c. e.
Secondary	rb IgG	gt	polyclonal	HRP	ProteinSimple	042-206	ready-to-use	c. e.
Secondary	ms IgG	gt	polyclonal	HRP	ProteinSimple	042-205	ready-to-use	c. e.
Secondary	rt IgG	dk	polyclonal	AF647	JacksonImmunoResearch	712-606-150	1:200	IF (tissue)
Secondary	rb IgG	gt	polyclonal	Cy3	JacksonImmunoResearch	111-165-003	1:200	IF (tissue)
Secondary	rb IgG	dk	polyclonal	Cy3	JacksonImmunoResearch	711-166-152	1:200	IF (cells)

Secondary	rt IgG	dk	polyclonal	Cy3	JacksonImmunoResearch	713-165-003	1:200	IF (tissue)
Secondary	sh IgG	dk	polyclonal	AF488	JacksonImmunoResearch	705-546-147	1:200	IF (cells)

dk: donkey, gt: goat; HRP: horseradish peroxidase; hu: human; ms: mouse; rb: rabbit, rt: rat, sh: sheep

Table S4 List of antibodies used for FACS/MACS

Immunogen	Host species	Clonality	Conjugate	Supplier	Catalog no.	Dilution
ms CD3	ha	monoclonal	PE-CF594	BD Biosciences	562286	1:67
ms CD11b	rt	monoclonal	BV605	BioLegend	101257	1:200
ms CD19	rt	monoclonal	APC-H7	BioLegend	115558	1:100
ms CD45	rt	monoclonal	AlexaFluor700	BD Biosciences	560510	1:50
ms CD49d	rt	monoclonal	BV421	BD Biosciences	740012	1:200
ms CD80	ha	monoclonal	BV650	BioLegend	104731	1:100
ms CD206	rt	monoclonal	FITC	BioLegend	141704	1:50
ms F4/80	rt	monoclonal	PE-Cy7	BioLegend	123114	1:50
ms Ly-6C	rt	monoclonal	BB790	BD Biosciences	624296	1:100
ms Ly-6G	rt	monoclonal	APC-Cy7	BioLegend	127624	1:67
ms MHC II	rt	monoclonal	APC	BioLegend	107614	1:50
ms P2RY12	rt	monoclonal	PE	BioLegend	848004	1:50

ha: hamster, ms: mouse, rt: rat

Table S5 List of primers used for quantitative real-time RT-PCR

Gene	Forward primer sequence (5′-3′)	Reverse primer sequence (5′-3′)
Actb	CGGTTCCGATGCCCTGAGGCTCTT	CGTCACACTTCATGATGGAATTGA
Kcnj2	CCCCTTTGTAGTGCCAGAGA	TTGCCTGGTTGTGGAGATCT
Nfat5	GGGGGTCAAACGACGAGATT	ACTGTCCGCACAACATAGGG
Pecam1	CCAAAGCCAGTAGCATCATGGTC	GGATGGTGAAGTTGGCTACAGG
Rps12	GAAGCTGCCAAAGCCTTAGA	AACTGCAACCAACCACCTTC
Slc6a6	AAAATGGTGGAGGTGCGTTC	ACACAATGACGATGGATGCG

Actb: actin beta; *Kcnj2*: potassium inwardly rectifying channel subfamily J member 2; *Nfat5*: nuclear factor of activated T cells 5; *Pecam1:* platelet-endothelial cell adhesion molecule 1; *Rps12*: 40S ribosomal protein S12; *Slc6a6*: solute carrier family 6 member 6

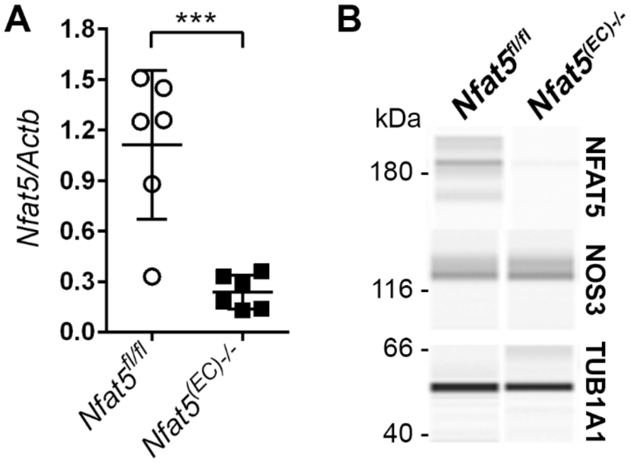

S1: Analysis of *Nfat5* expression in BEC isolated from mouse brains. Single-cell suspensions were prepared from mouse brains through mechano-enzymatic tissue digestion followed by MACS-based ($CD45^{low}$/$CD146^{high}$) BEC isolation. **(A)** Real-time RT-PCR was used to analyze gene expression in freshly isolated BEC from $Nfat5^{fl/fl}$ and $Nfat5^{(EC)-/-}$ mice. Values were normalized to *Actb* and expressed as fold change of $Nfat5^{fl/fl}$ (n=6 per group; unpaired two-tailed Student's t test; *** p<0.001). **(B)** Exemplary analysis of lysates from MAC-sorted BEC by capillary electrophoresis/immunodetection showing a decrease in NFTA5 abundance in BEC derived from $Nfat5^{(EC)-/-}$ mice (NOS3 level were not altered, TUBA1A (α-tubulin) served as reference).

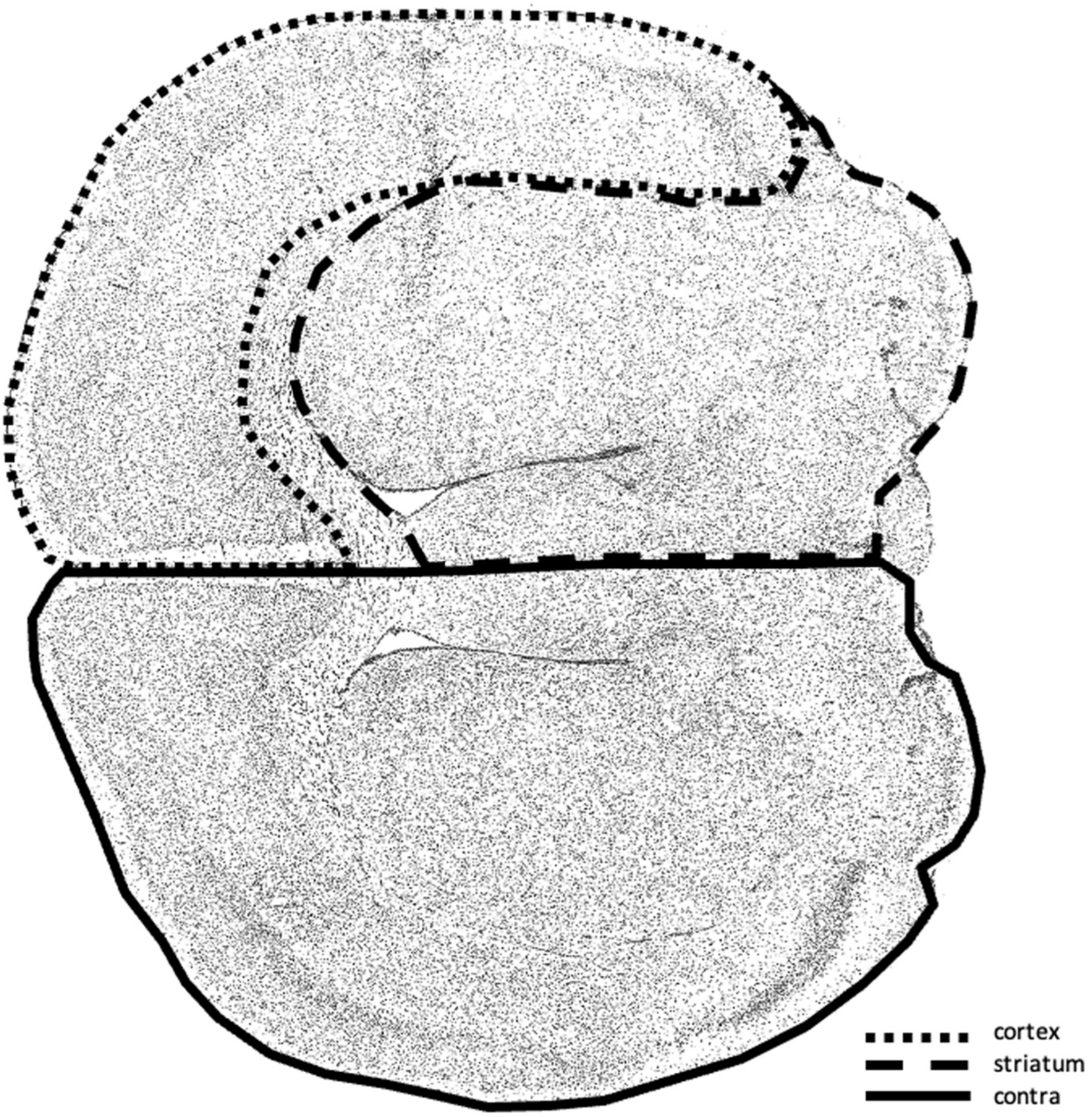

S2: Definition of anatomical ROIs for immunofluorescence analyses. Allen mouse brain atlas (https://mouse.brain-map.org/static/atlas) served as reference to determine anatomical sub-regions for immunofluorescence analyses. Regions of interest were defined as the cortex, the subcortical region consisting almost completely of the striatum and the contralateral hemisphere.

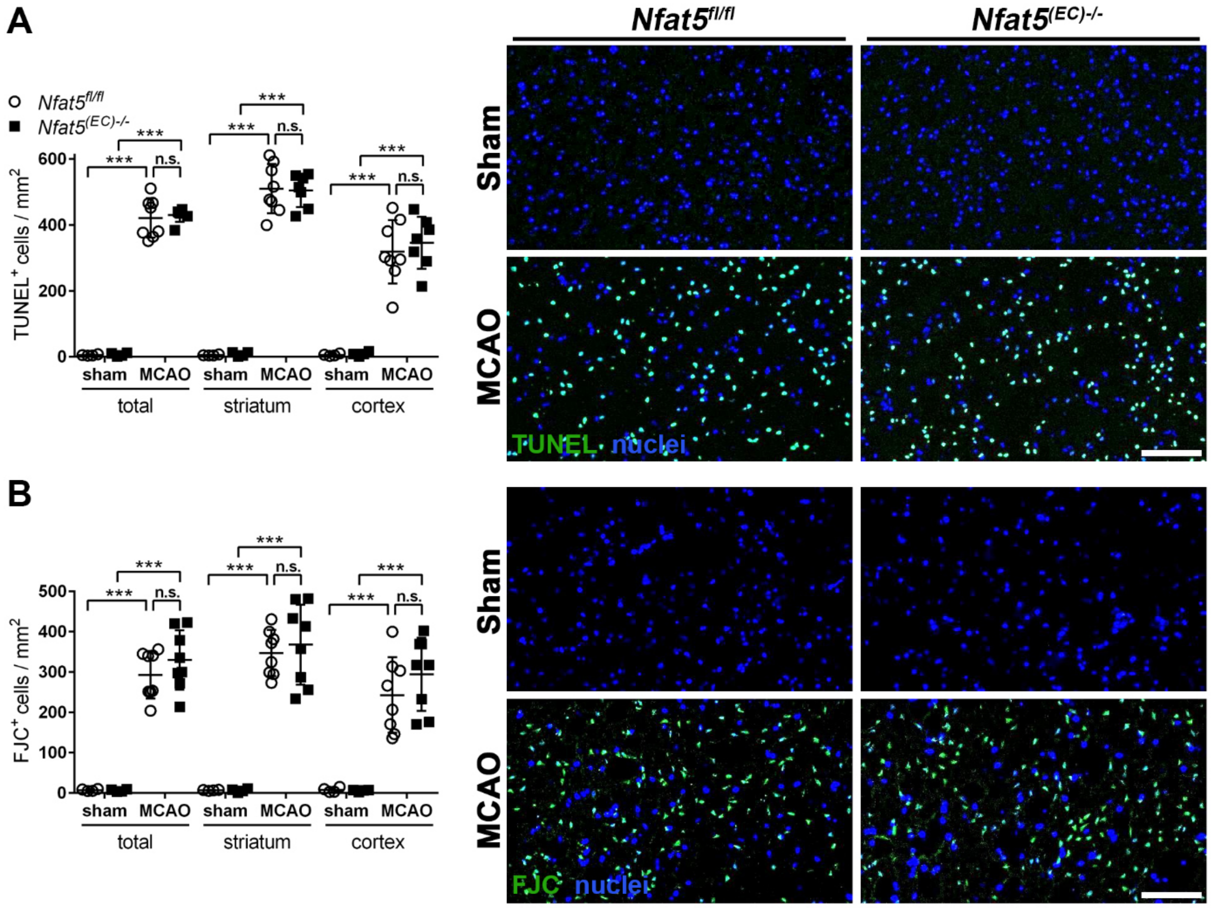

S3: Endothelial cell-specific knockout of *Nfat5* does not affect brain cell apoptosis and neuronal degeneration during acute ischemic stroke. $Nfat5^{(EC)-/-}$ and $Nfat5^{fl/fl}$ mice were subjected to 45 min of MCAO followed by 24 h reperfusion. Sham-operated mice served as control. **(A)** TUNEL method was applied to quantify brain cell apoptosis. (n=4-8 per group; Two-way ANOVA with Holm-Sidak's multiple comparisons test; *** p<0.001). Representative microphotographs: TUNEL (green), DAPI (blue). Scale bar: 100 µm. **(B)** FJC staining has been used to label degenerating neurons. (n=4-8 per group; Two-way ANOVA with Holm-Sidak's multiple comparisons test; *** p<0.001). Representative microphotographs: FJC (green), DAPI (blue). Scale bar: 100 µm.

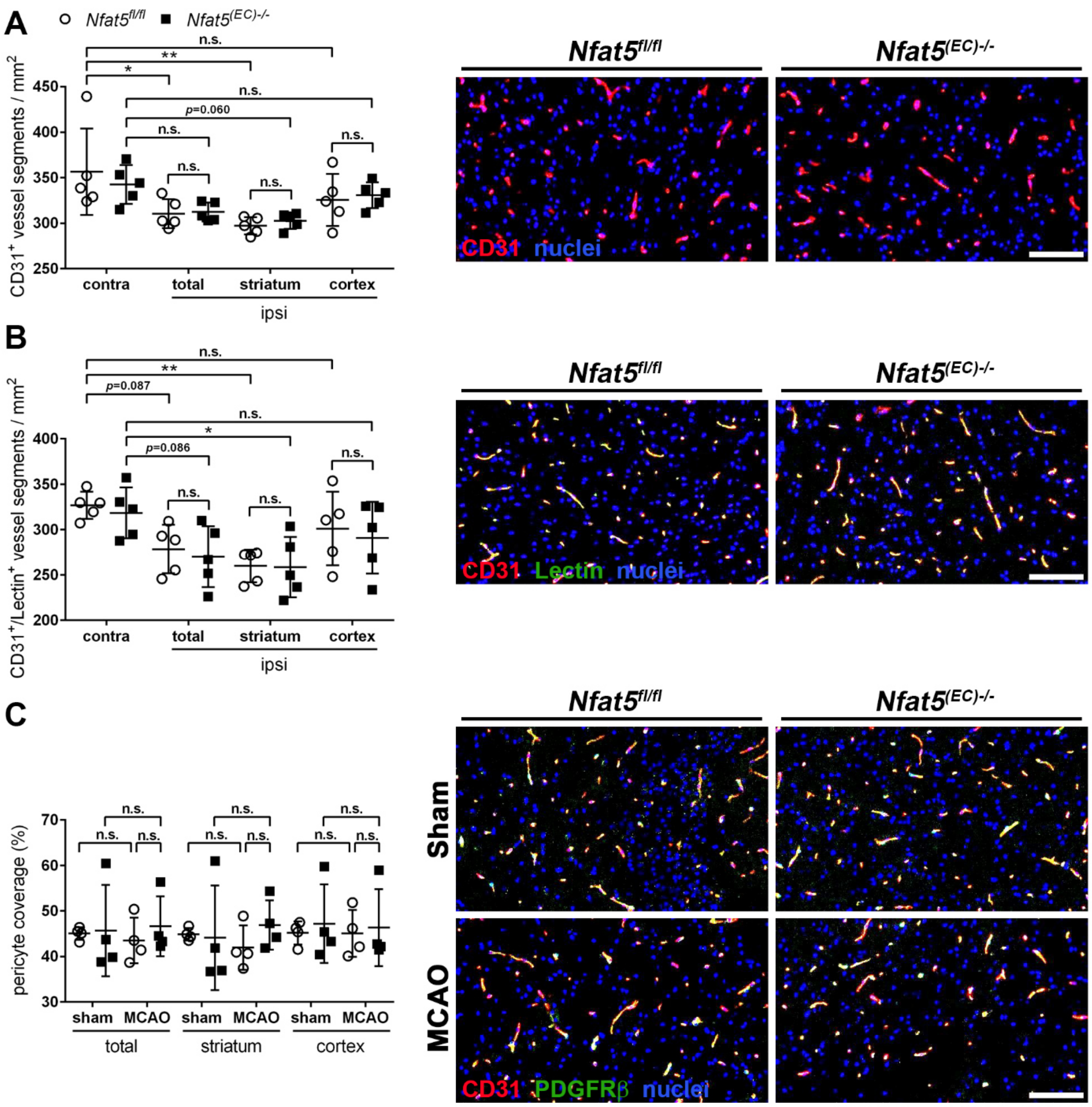

S4: Endothelial cell-specific knockout of *Nfat5* does not influence the cerebrovascular integrity during acute ischemic stroke. *Nfat5*$^{(EC)-/-}$ and *Nfat5*$^{fl/fl}$ mice were subjected to 45 min of MCAO followed by 24 h reperfusion. Sham-operated mice served as control. **(A, B)** For evaluation of perfused cerebrovasculature, DyLight 488 conjugated tomato lectin was intravenously applied. The density of **(A)** total vessel segments and **(B)** lectin-positive perfused vessel segments across total contralateral and ipsilateral brain hemispheres of mice subjected to I/R injury was determined by CD31 immunofluorescence staining (n=5 per group; Two-way ANOVA with Holm-Sidak's multiple comparisons test; * p<0.05, ** p<0.01, *** p<0.001). Representative microphotographs: CD31 (red), lectin (green), DAPI (blue). Scale bar: 100 µm. **(C)** Cerebrovascular pericyte coverage was estimated by IF-based quantification of PDGFRβ+/CD31+ area related to total CD31+ area (n=4 per group; Two-way ANOVA with

Holm-Sidak's multiple comparisons test). Representative microphotographs: CD31 (red), PDGFRβ+ (green), DAPI (blue). Scale bar: 100 µm.

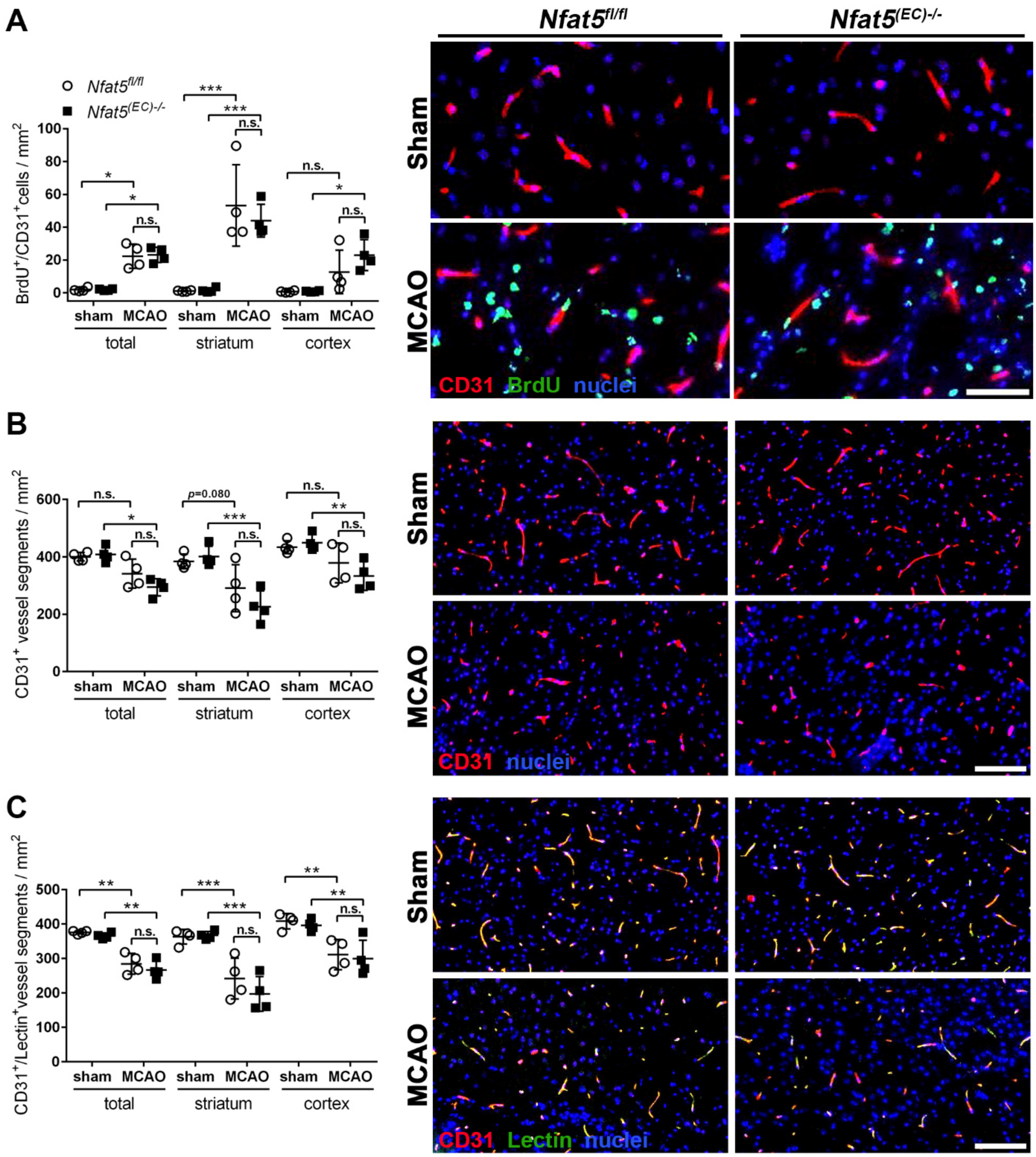

S5: Endothelial cell-specific knockout of *Nfat5* does not affect compensatory angiogenesis in response to ischemic stroke. *Nfat5*$^{(EC)-/-}$ and *Nfat5*$^{fl/fl}$ mice were subjected to 45 min of MCAO followed by 28 d reperfusion. Sham-operated mice served as control. **(A)** BrdU (50 mg/kg) was applied intraperitoneally to mice once daily for 4 consecutive days from day 4 to 7 post-MCAO for labeling of proliferating cells. BrdU/CD31 co-immunofluorescence staining was used to quantify proliferating endothelial cells (*n*=4 per group; Two-way ANOVA with

Holm-Sidak's multiple comparisons test; * $p<0.05$, *** $p<0.001$). Representative microphotographs: BrdU (green), CD31 (red), DAPI (blue). Scale bar: 50 μm. **(B, C)** For evaluation of perfused cerebrovasculature, DyLight 488 conjugated tomato lectin was intravenously applied. The density of **(B)** total vessel segments and **(C)** lectin-positive perfused vessel segments across the ipsilateral brain hemisphere was determined by CD31 immunofluorescence staining ($n=4$ per group; Two-way ANOVA with Holm-Sidak's multiple comparisons test; * $p<0.05$, ** $p<0.01$, *** $p<0.001$). Representative microphotographs: CD31 (red), lectin (green), DAPI (blue). Scale bar: 100 μm.

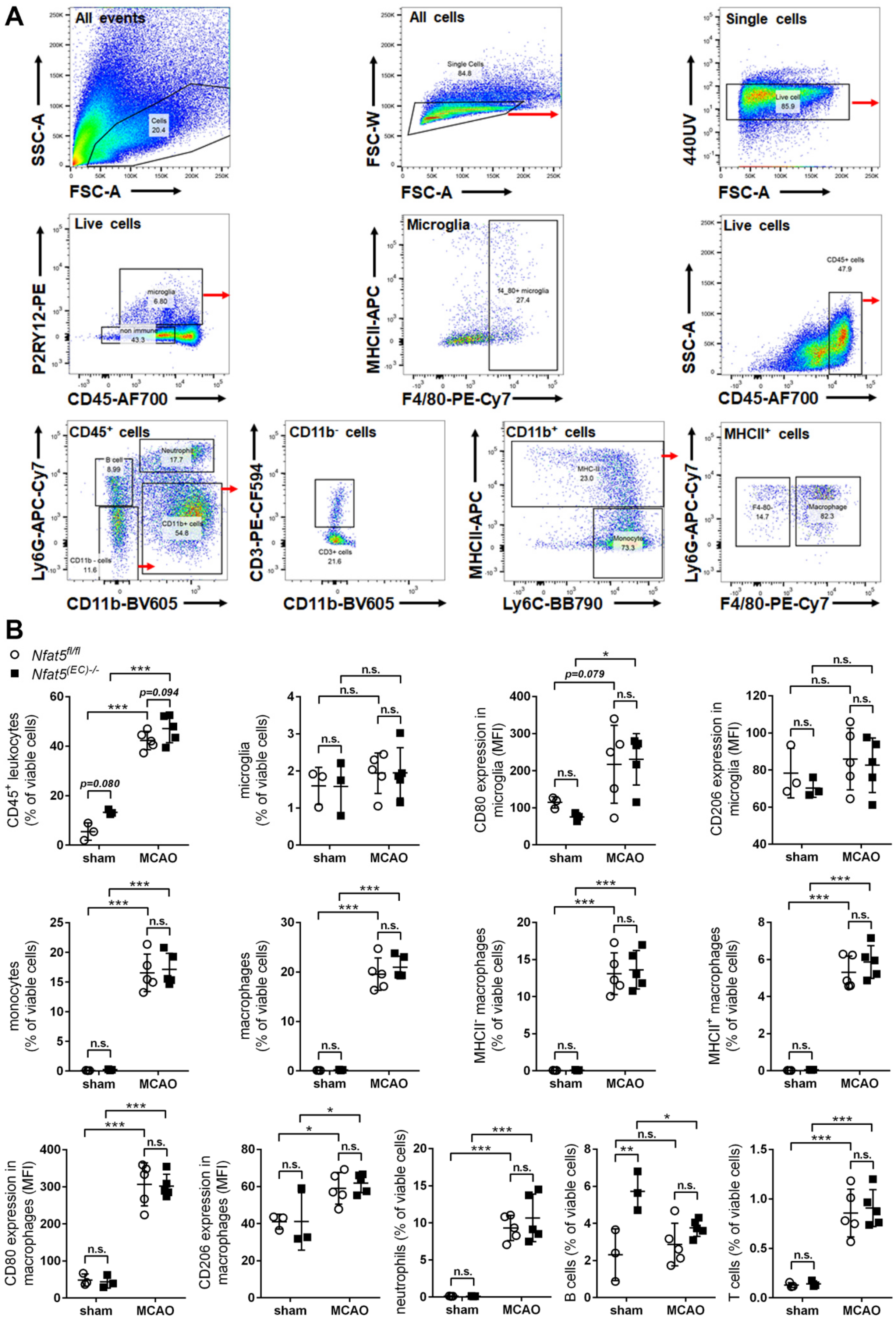
A
All events
SSC-A
FSC-A
Cells 20.4
All cells
FSC-W
Single Cells 84.8
Single cells
440UV
Live cell 85.9
Live cells
P2RY12-PE
CD45-AF700
microglia 6.80
non immune 43.3
Microglia
MHCII-APC
F4/80-PE-Cy7
f4_80+ microglia 27.4
CD45+ cells 47.9
CD45+ cells
Ly6G-APC-Cy7
CD11b-BV605
Neutrophils 17.7
B cell 8.99
CD11b+ cells 54.8
CD11b - cells 11.6
CD11b- cells
CD3-PE-CF594
CD3+ cells 21.6
CD11b+ cells
MHC-II 23.0
Monocytes 73.3
Ly6C-BB790
MHCII+ cells
F4-80- 14.7
Macrophage 82.3
B
Nfat5fl/fl
Nfat5(EC)-/-
CD45+ leukocytes (% of viable cells)
p=0.080
p=0.094
microglia (% of viable cells)
CD80 expression in microglia (MFI)
p=0.079
CD206 expression in microglia (MFI)
monocytes (% of viable cells)
macrophages (% of viable cells)
MHCII- macrophages (% of viable cells)
MHCII+ macrophages (% of viable cells)
CD80 expression in macrophages (MFI)
CD206 expression in macrophages (MFI)
neutrophils (% of viable cells)
B cells (% of viable cells)
T cells (% of viable cells)
sham
MCAO
n.s.

S6: Endothelial cell-specific knockout of *Nfat5* does not influence recruitment and polarization of brain-resident and circulating immune cells during acute ischemic stroke. *Nfat5*$^{(EC)-/-}$ and *Nfat5*$^{fl/fl}$ mice were subjected to 45 min of MCAO followed by 24 h reperfusion. Sham-operated mice served as control. Single-cell suspensions were prepared from the ipsilateral brain hemisphere. Flow cytometry was applied to determine number and polarization of brain-resident microglia and infiltrating leukocytes. **(A)** Representative plots showing the gating strategy for flow cytometric analysis of CD45^{+} cells including microglia (P2RY12^{+}/CD49d^{-}), monocytes (CD11b^{+}/Ly6C^{+}/MHCII^{-}), macrophages (CD11b^{+}/Ly-6C^{+}/F4/80^{+}/MHCII^{+}; Mφ), neutrophils (CD11b^{+}/Ly-6G^{+}), B cells (CD11b^{-}/CD19^{+}) and T cells (CD11b^{-}/CD3^{+}) in brains of mice subjected to experimental ischemic stroke. CD80 against CD206 plotting in microglia and macrophages showed only one cell population. Therefore, the median (midpoint of the data) was calculated. **(B)** Immune cell populations (shown as a percentage of viable cells) as well as expression of polarization markers (CD80, CD206; determined as median fluorescence intensity (MFI)) in microglia and macrophages isolated from brain parenchyma of mice subjected to either sham surgery or MCAO (n=3-5 per group; Two-way ANOVA with Holm-Sidak's multiple comparisons test; * p<0.05, ** p<0.01, *** p<0.001).

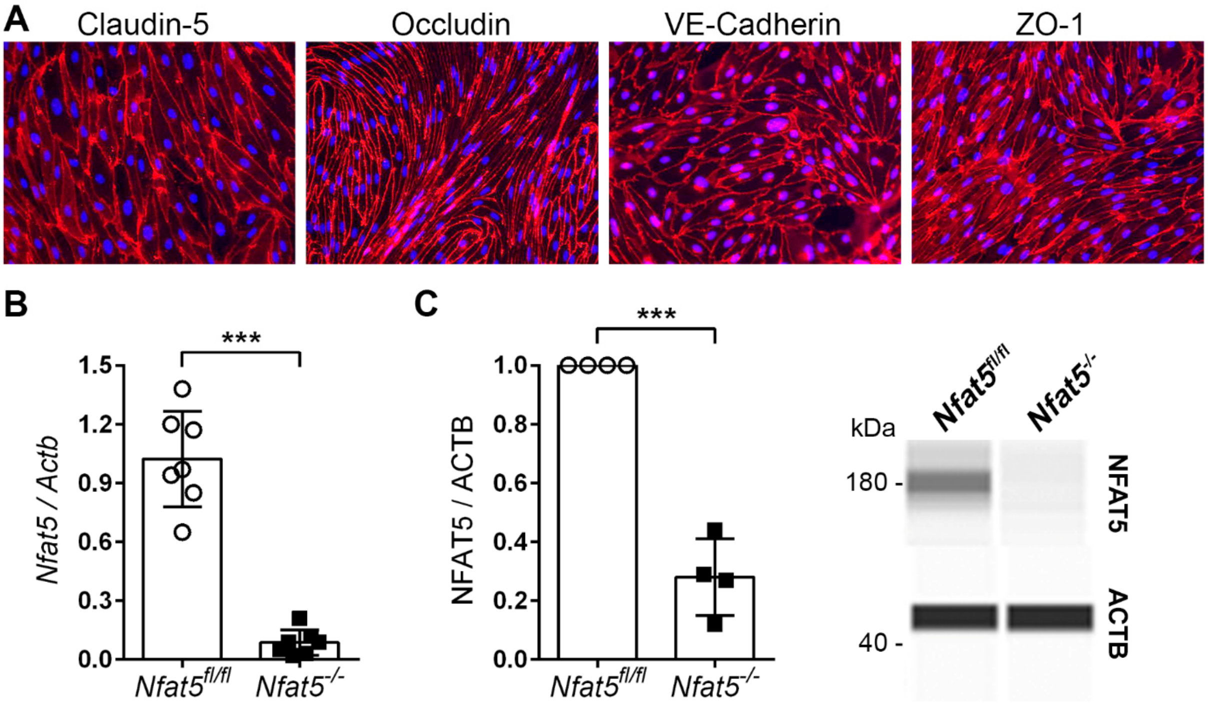

S7: Characterization of *Nfat5* knockout generated by using the CreERT2/loxP recombination system in cultured BEC. Primary cultures of BEC were established from microvessels isolated from adult *Nfat5*$^{fl/fl}$ mice. **(A)** Immunofluorescence analyses confirmed sustained expression of tight and adherens junction proteins in cultured BEC. **(B, C)** *Nfat5*$^{fl/fl}$ BEC were exposed to 1 µM 4-hydroxytamoxifen (4-HT; *Nfat5*$^{-/-}$) or solvent for 3 d followed by a recovery period of 3 d. Real-time RT-PCR **(B)** and capillary electrophoresis/immunodetection **(C)** showed a significant decrease in *Nfat5* expression and NFAT5 protein level after 4-HT treatment. Values were normalized to β-actin (ACTB) and expressed as fold change of control (*n*=4-7 per group; unpaired two-tailed Student's t test; *** $p<0.001$).

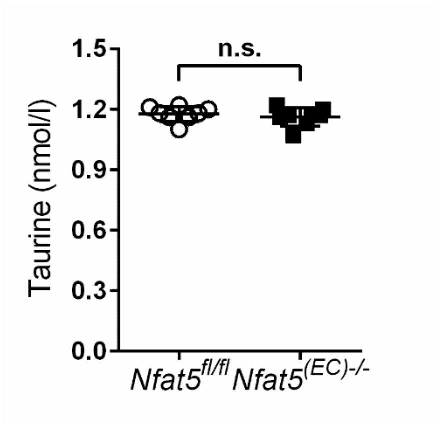

S8: Determination of the concentration of taurine in brain tissue. PBS-perfused brain hemispheres of *Nfat5*$^{fl/fl}$ and *Nfat5*$^{(EC)-/-}$ were lysed. Taurine concentration was assessed by applying a mouse-specific taurine ELISA (MBS756005, MyBioSource, USA) according to manufacturer's instructions (*n*=8 per group; unpaired two-tailed Student's t test).

Kcnj2_PM001026021
(mouse chr11)

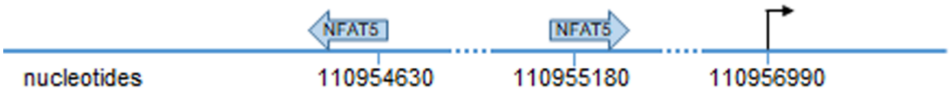

Matrix	Factor name	Position (strand)	Core score	Matrix score	Sequence
V$NFAT5_Q5_01	NFAT5	110954619 (-)	1.000	0.965	gtagagaTTTCC
V$NFAT5_Q5_01	NFAT5	110955176 (+)	1.000	0.977	GGAAAactcttc

S9: In silico analysis of the *Kcnj2* promoter. NFAT5 binding sites were predicted in the promoter for mouse *Kcnj2* PM001026021 (mouse chr11, genomic coordinates: build mm39/GRCm39: Chr11:110946990..110957990 +) by using the geneXplain platform for systems biology (www.genexplain.com) and MATCH (Kel, Alexander E., et al. "MATCH^{TM}: a tool for searching transcription factor binding sites in DNA sequences." Nucleic acids research 31 (2003): 3576-3579) according to the TRANSFAC database release 2023.2. The analyzed *Kcnj2* promoter region covered −5000 to +100 bp relative to the transcriptional start site. We used the cut-off "minSUM" to minimize false negative and false positive matches. The matrix similarity score describes the match quality between the NFAT5 matrix and the analyzed promoter sequence. The core similarity score denotes the match quality of the core sequence of the NFAT5 matrix (defined as the five most conserved consecutive positions within a matrix). Capital letters indicate the positions in the sequence that match with the core sequence of the matrix.

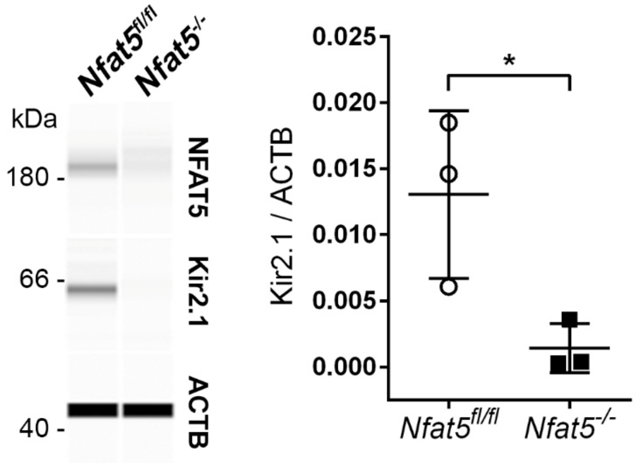

S10: Detection of Kir2.1 in cultured BEC.

BEC were exposed to 4-hydroxytamoxifen (*Nfat5*$^{-/-}$) or solvent (*Nfat5*$^{fl/fl}$). Capillary electrophoresis/immunodetection showed a significant decrease in Kir2.1 protein level after NFAT5 knockout. β-actin (ACTB) was used as reference (n=3; unpaired two-tailed Student's t test; * p<0.05).

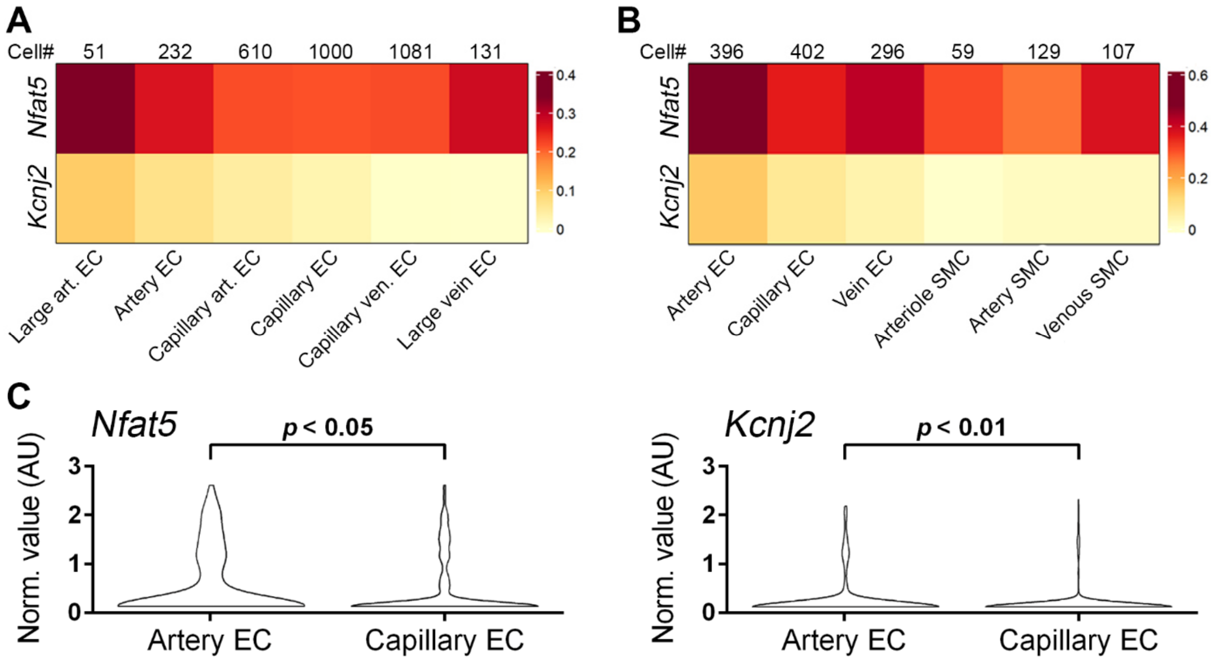

S11: Analyses of *Nfat5* and *Kcnj2* expression in BEC. Metaanalysis of scRNA-seq data was performed by using BBrowser (Bbrowser version 3.5.26, Bioturing). (**A**) Box plot showing the relative expression level of *Nfat5* and *Kcnj2* of mouse (C57BL6) brain endothelial cell (EC) populations as defined by the authors of study ID: E-MTAB-8077; Kalucka et al., Cell 180, 764-779.e20 (2020). (**B**) Box plot showing the relative expression level of *Nfat5* and *Kcnj2* of mouse (C57BL6) brain endothelial and smooth muscle (SMC) cell populations as defined by the authors of study ID: vanlandewijck2018_brain_lung; Vanlandewijck, M. et al., Nature 554, 475–480 (2018). (**C**) Violin plots comparing the *Nfat5* and *Kcnj2* expression level (height) and frequency (width) of mouse brain artery and capillary EC (study ID: study ID: vanlandewijck2018_brain_lung, *p*-values (FDR) as indicated, One-Way-ANOVA).

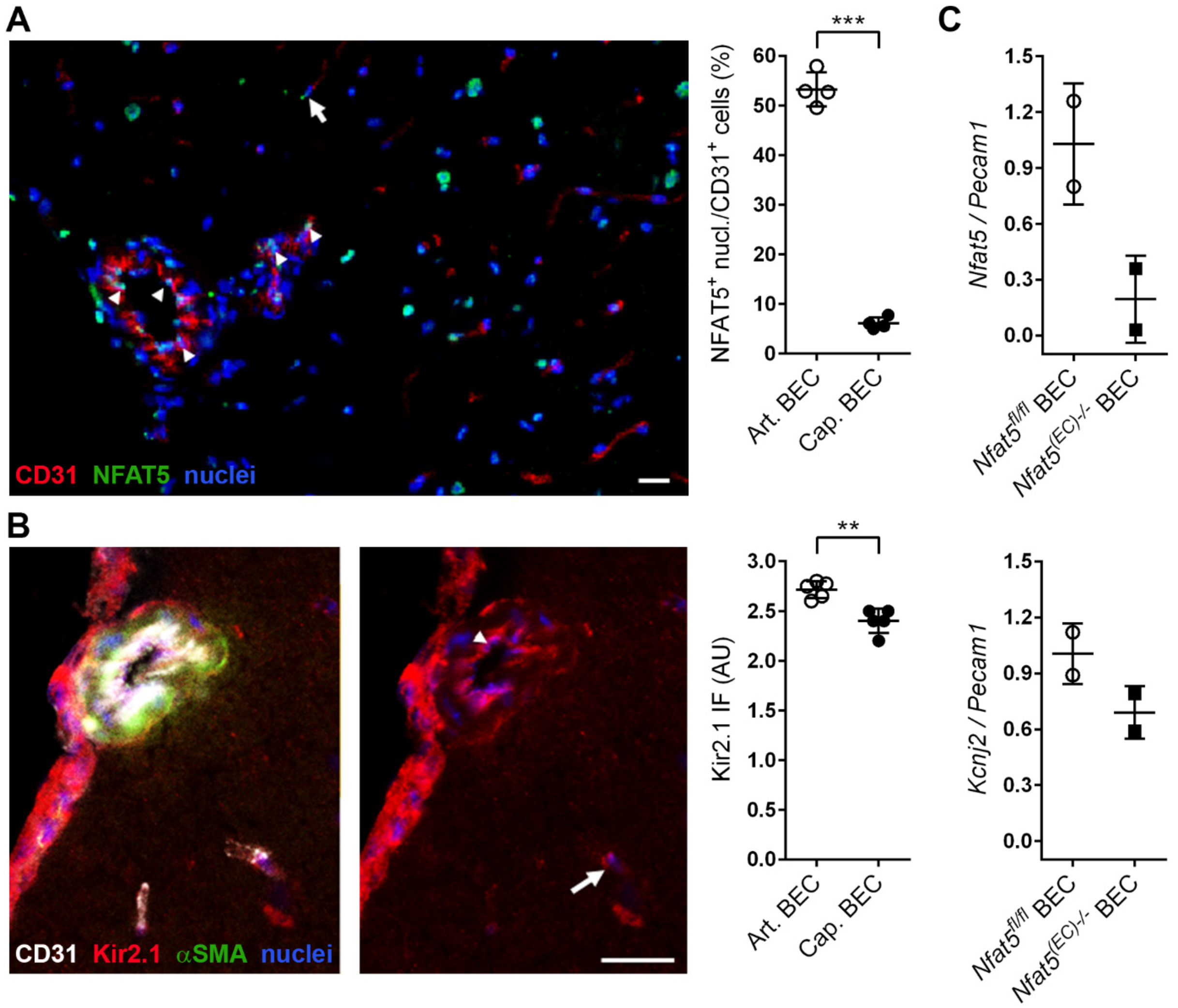

S12: Analysis of *Nfat5* and *Kcnj2* expression in BEC. (A) Immunofluorescence microscopy showing NFAT5 in the nuclei of an artery (arrowheads). The arrow points to a nucleus of a capillary (scale bar: 20 µm). The percentage of CD31-positive endothelial cells with NFAT5-positive nuclei was determined in brains of *Nfat5*$^{fl/fl}$ mice (n=4 per group; unpaired two-tailed Student's t test; *** p<0.001 as indicated). **(B)** Immunofluorescence microscopy showing the localization of Kir2.1 (red fluorescence) in endothelial cells of an artery (arrowhead) and a capillary (arrow) in a *Nfat5*$^{fl/fl}$ mouse brain (scale bar: 20 µm). The Kir2.1-specific fluorescence in CD31^{+} arterial and capillary ECs in *Nfat5*$^{fl/fl}$ mouse brains was graded by semiquantitative assessment of the signal intensity (0= no signal, 1 = low, 2 = mean, 3 = high; n=5 per group; unpaired two-tailed Student's t test; ** p<0.01 as indicated). **(C)** Single-cell suspensions were prepared from brain hemispheres of *Nfat5*$^{fl/fl}$ and *Nfat5*$^{(EC)-/-}$ mice through mechano-enzymatic tissue digestion followed by MACS-based (CD45^{low}/CD146^{high}) BEC isolation (includes all types of EC). Real-time RT-PCR was used to exemplarily analyze *Nfat5* and *Kcnj2* gene expression. Values were normalized to *Pecam1* and expressed as fold change of *Nfat5*$^{fl/fl}$ (n=2 per group).

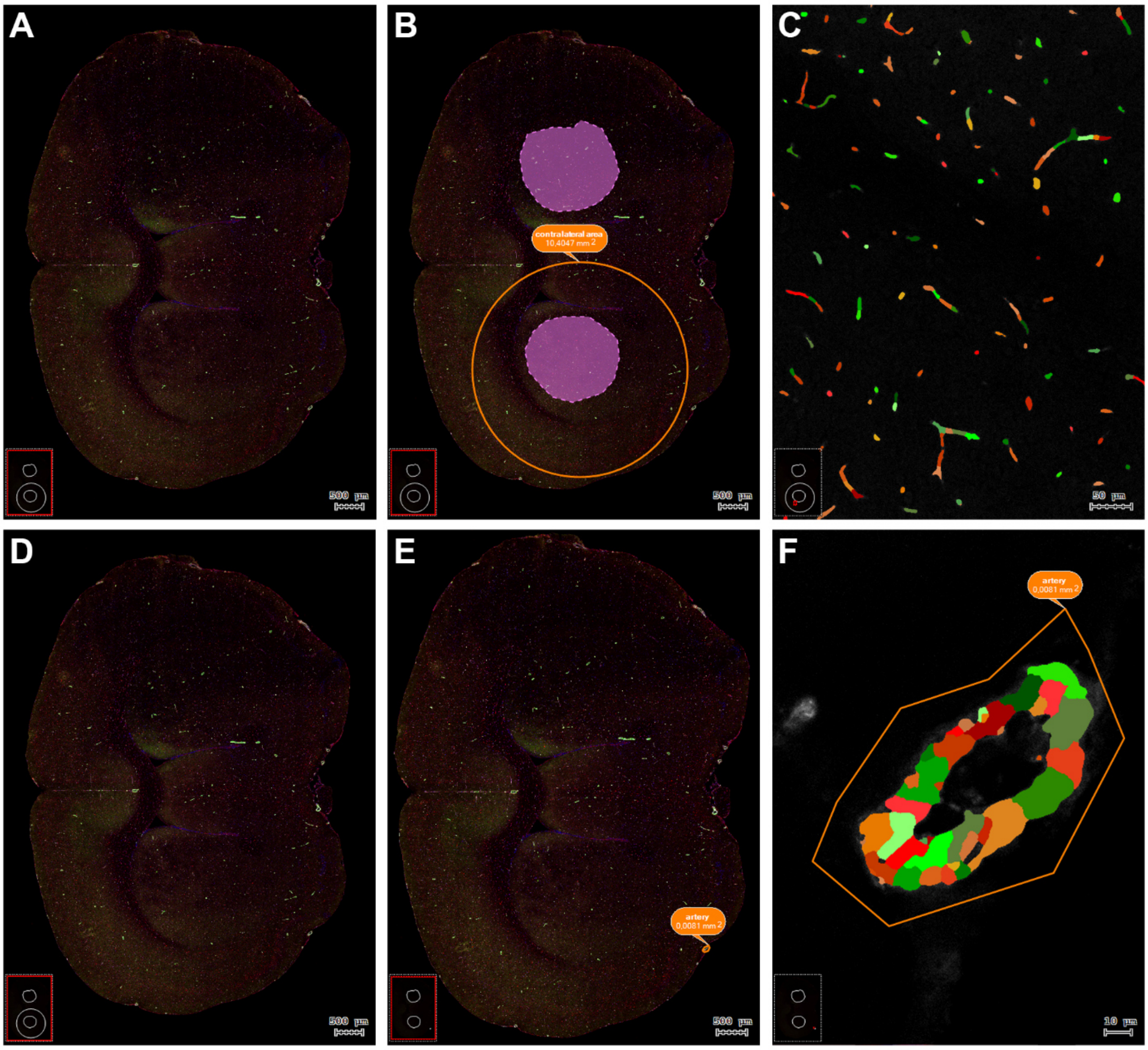

S13: Overview illustrating the automated detection of CD31^{+} endothelial cells of capillaries within the estimated stroke area in murine brain sections and arterial branches originating from the middle cerebral artery. **(A)** Overview of brain section (immunofluorescence-based detection of Kir2.1 (red), CD31 (white), αSMA (green) and nuclei (blue)). **(B)** Region of interest (ROI, orange circle), encircling the estimated stroke area and excluding the core stroke area (purple). **(C)** Automated detection of CD31^{+} ECs (colors indicate the segmentation of the detected EC population). **(D)** Overview of brain section (immunofluorescence-based detection of Kir2.1 (red), CD31 (white), αSMA (green) and nuclei (blue)). **(E)** Region of interest (ROI, orange circle), encircling an artery. **(F)** Automated detection of CD31^{+} ECs (colors indicate the segmentation of the detected EC population).

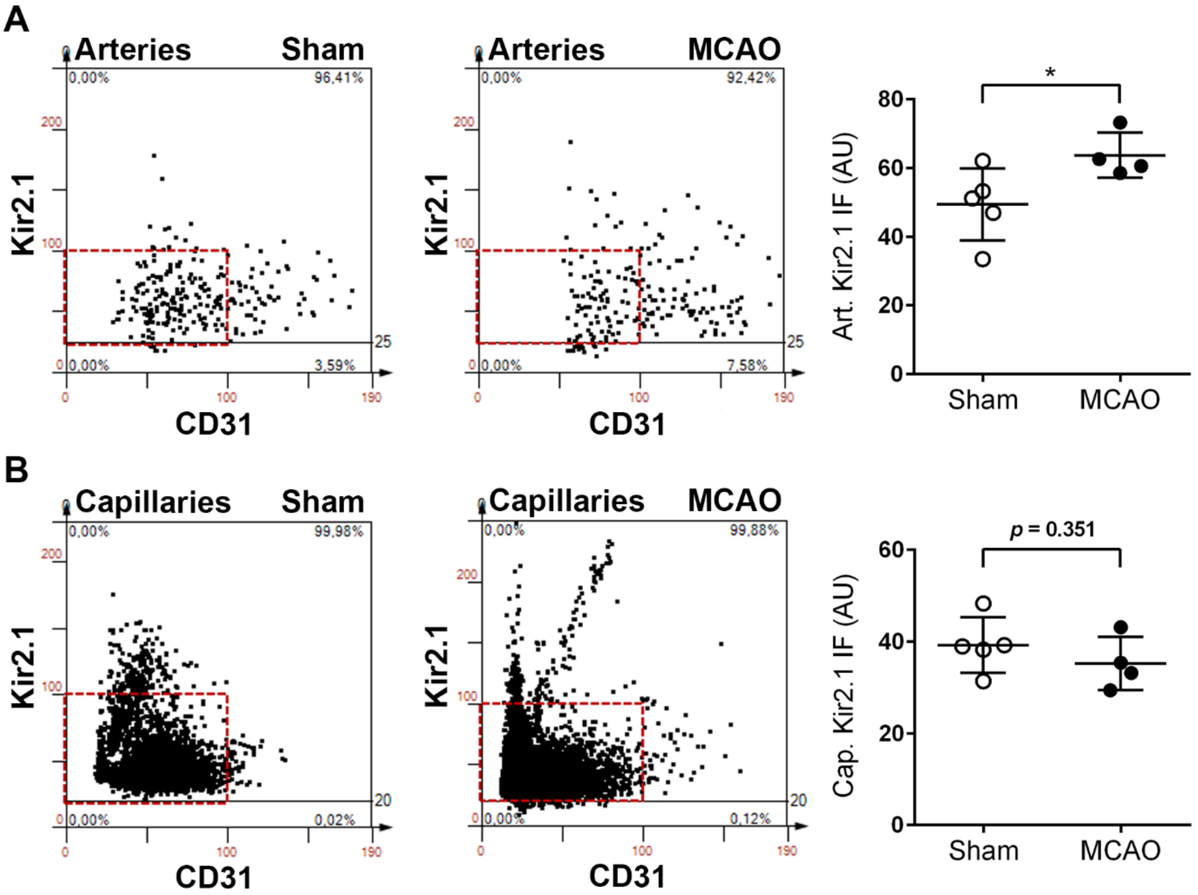

S14: Automated analysis of the Kir2.1-specific fluorescence intensity in CD31^{+} capillary and arterial endothelial cells. Representative scatter plots showing the fluorescence intensity of Kir2.1 (y-axis) and CD31 (x-axis) of individual arterial (**A**) and capillary (**B**) endothelial cells in brains from *Nfat5$^{fl/fl}$* mice exposed to control conditions (sham) or after MCAO (24 h reperfusion). Values shown top and bottom right within the plots indicate the percentage of cells detected above and below the ROI-specific fluorescence background levels (indicated the black line) for CD31^{+}EC detection. The graphs summarize the mean Kir2.1-specific fluorescence values of arterial (**A**) and capillary (**B**) endothelial cells in selected ROIs (*n*=4-7 per group; unpaired two-tailed Student's t test; * *p*<0.05 as indicated).

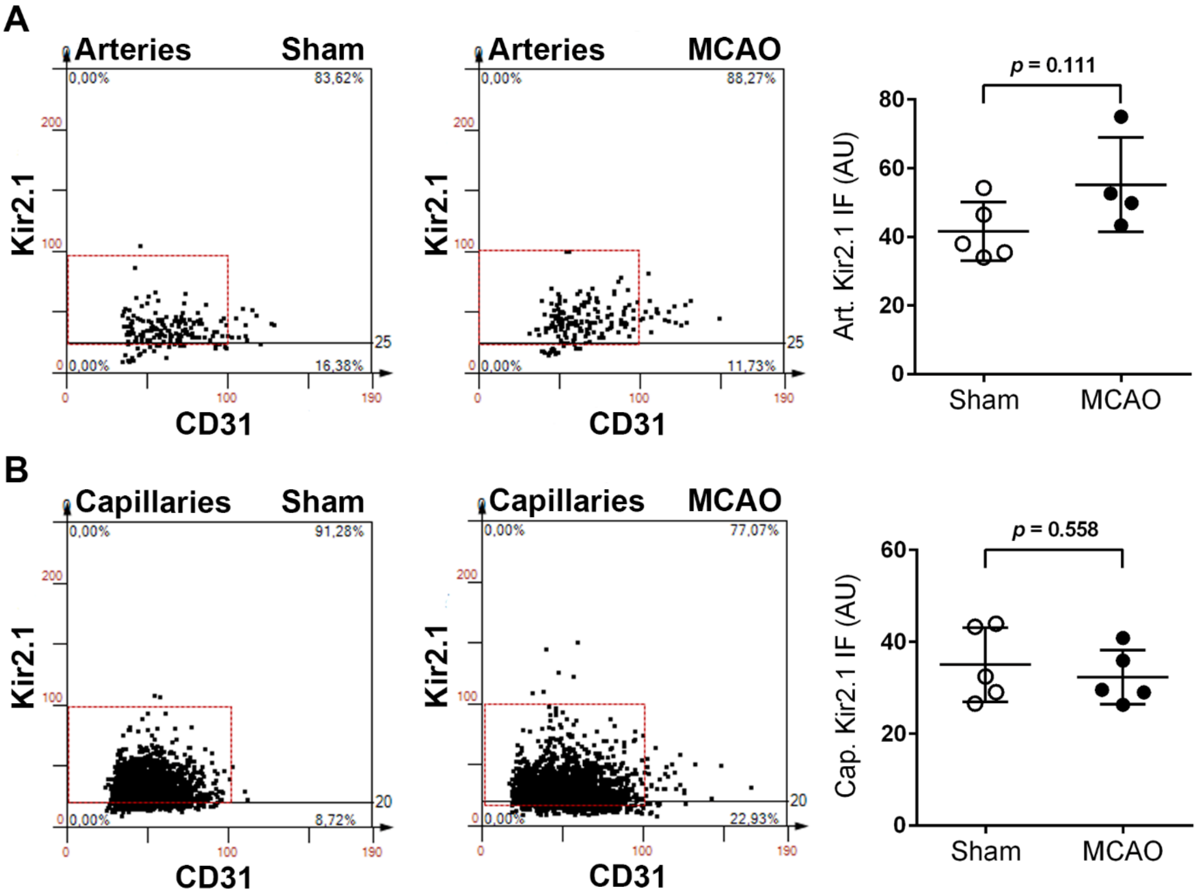

S15: Automated analysis of the Kir2.1-specific fluorescence intensity in $CD31^+$ capillary and arterial endothelial cells of *Nfat5*$^{(EC)-/-}$ mice. Representative scatter plots showing the fluorescence intensity of Kir2.1 (y-axis) and CD31 (x-axis) of individual arterial (**A**) and capillary (**B**) endothelial cells in brains from *Nfat5*$^{(EC)-/-}$ mice exposed to control conditions (sham) or after MCAO (24 h reperfusion). Values shown top and bottom right within the plots indicate the percentage of cells detected above and below the ROI-specific fluorescence background levels (indicated the black line) for $CD31^+$EC detection. The graphs summarize the mean Kir2.1-specific fluorescence values of arterial (**A**) and capillary (**B**) endothelial cells in selected ROIs (*n*=4-5 per group; unpaired two-tailed Student's t test). Note the difference to the scatter plots shown in S14 for *Nfat5*$^{fl/fl}$ mice after MCAO.
